# Genome-Wide Comparison of *Magnaporthe* Species Reveals a Host-Specific Pattern of Secretory Proteins and Transposable Elements

**DOI:** 10.1371/journal.pone.0162458

**Published:** 2016-09-22

**Authors:** Meghana Deepak Shirke, H. B. Mahesh, Malali Gowda

**Affiliations:** 1 Genomics Laboratory, Centre for Cellular and Molecular Platforms, National Centre for Biological Sciences, Bengaluru-560065, India; 2 Manipal University, Manipal-576104, India; 3 Marker Assisted Selection Laboratory, Department of Genetics and Plant Breeding, University of Agricultural Sciences, Bengaluru- 560065, India; 4 Genomics Discovery Program, School of Conservation, Life Science and Health Sciences, TransDisciplinary University, Foundation of Revitalization of Local Health Traditions, Bengaluru- 560064, India; National Institute of Plant Genome Research, INDIA

## Abstract

Blast disease caused by the *Magnaporthe* species is a major factor affecting the productivity of rice, wheat and millets. This study was aimed at generating genomic information for rice and non-rice *Magnaporthe* isolates to understand the extent of genetic variation. We have sequenced the whole genome of the *Magnaporthe* isolates, infecting rice (leaf and neck), finger millet (leaf and neck), foxtail millet (leaf) and buffel grass (leaf). Rice and finger millet isolates infecting both leaf and neck tissues were sequenced, since the damage and yield loss caused due to neck blast is much higher as compared to leaf blast. The genome-wide comparison was carried out to study the variability in gene content, candidate effectors, repeat element distribution, genes involved in carbohydrate metabolism and SNPs. The analysis of repeat element footprints revealed some genes such as naringenin, 2-oxoglutarate 3-dioxygenase being targeted by *Pot*2 and Occan, in isolates from different host species. Some repeat insertions were host-specific while other insertions were randomly shared between isolates. The distributions of repeat elements, secretory proteins, CAZymes and SNPs showed significant variation across host-specific lineages of *Magnaporthe* indicating an independent genome evolution orchestrated by multiple genomic factors.

## Introduction

The blast disease, which is caused by an Ascomycetes fungal pathogen *Magnaporthe* affects the productivity of important food crops like rice, wheat and finger millet. The *Magnaporthe* species complex consists of five species, *M*. *grisea*, *M*. *oryzae*, *M*. *salvinii*, *M*. *poae* and *M*. *rhizophila*. The causative agent of the blast, *Magnaporthe oryzae* is the most destructive member of the *Magnaporthe* species complex, with a wide host range. Blast has been reported in more than 80 countries across the globe and the pathogen has been known to infect more than 130 host species of the Poaceae family [1]. This pathogen can infect at various stages of a crop plant growth including leaf, stem, neck, collar, node and root. The broad host range, continuous genetic variation, evolution and host shifts are the main reasons behind the emergence of virulent pathotypes of *Magnaporthe*, which make blast management a daunting task.

The blast fungus has a long history of evolution with the first occurrence reported on rice in 1637 [2]. The fungus has undergone multiple host shifts on various host species. The most recent host shift on cultivated crops was reported on wheat in 1985 in the Parana state of Brazil, causing a severe yield loss [3]. In addition to wheat blast, finger millet blast is also a serious constraint in Asian and African countries [4]. The fungus is also known to infect co-cultivated grass species in the absence of primary host cultivation as an adaptive strategy [5]. Despite such wide host range, the majority of blast pathogens exists as host-specific forms, whose virulence spectrum is limited to a particular host species. Previous reports on host-specificity indicated the presence of certain host factors, and the loss of avirulence genes and effector proteins in maintaining the host-specific population of *Magnaporthe*. However, the detailed mechanism underlying the maintenance of host specificity is largely unknown.

Our previous studies on the whole genome comparison among *Magnaporthe* field isolates of rice have shown ample genetic variation in terms of SNPs, INDELs and gene content [6]. We extended our sequencing efforts further, to understand the genetic constellation and variation between *Magnaporthe* isolates infecting rice (*Oryza sativa* L.), finger millet (*Eleusine coracana* L. Gaertner), foxtail millet (*Setaria italica* L.) and buffel grass (*Cenchrus ciliaris* L.). Because understanding the mechanism of host adaptation is necessary to predict the reciprocation of plant pathogens to changing agro-climatic conditions, the emergence of new diseases due to the expansion of host boundaries, and breakdown of existing resistance. The isolates were analyzed extensively to compare the core genome, gene content, gene family duplications, repeat element footprints and distribution, carbohydrate-active enzymes (CAZymes), candidate effectors, single nucleotide polymorphism and genes under positive selection. This is the first whole genome study of tropical rice and non-rice *Magnaporthe* isolates from India, which will accelerate the understanding of the spectrum of fungal virulence and the mechanisms of host specificity.

## Materials and Methods

### Collection of the *Magnaporthe* field isolates and storage

The pure cultures of the *Magnaporthe* isolates from rice and non-rice host plants were obtained from blast infected leaf and neck tissues using water agar based single spore isolation method. The fungal isolates MG01 and MG02 were isolated from a HR-12 rice (*Oryza sativa* L.) variety from infected leaf and neck tissues, respectively. Another isolate MG10 was isolated from infected leaf tissue of T7 rice variety. The MG08 was isolated from infected leaf tissue of foxtail millet (*Setaria italica* L.). These (MG01, MG02, and MG08) isolates were collected from Mandya (12.5200°N, 76.9000°E) location. The fungal isolates MG03, MG04, MG12, MG05, and MG07 were collected from Bangalore (12.9667°N, 77.5667°E) location. These locations come under the purview of our institute hence, the special permissions were not required to conduct the experiment as per research ethics. The MG03, MG04, and MG12 were isolated from infected leaf and neck of tissues of finger millet (*Eleusine coracana* L. Gaertner) varieties Uduru Mallige (MG03 from leaf tissue, MG04 from neck tissue) and PR202 (MG12 from leaf tissue). Similarly, MG05 and MG08 were isolated from infected leaf tissues of foxtail millet and MG07 from buffel grass (*Cenchrus celiaris* L.). The oatmeal agar (M397-500G, Himedia) medium was used for growth and maintenance of the isolates. These isolates were stored on sterilized filter paper discs at -20°C for long-term storage.

### Cross-infectivity assay

For a cross infection assay, fungal isolates were inoculated on four varieties (HR-12, Co-39, Tetep and Tadukan) of rice, three varieties (Uduru Mallige, GPU48, and GPU28) of finger millet and buffel grass. Rice varieties HR-12, Co-39 (susceptible to blast disease), and Tetep, Tadukan (resistant to blast disease) were obtained from the Indian Institute of Rice Research (IIRR), Hyderabad. The finger millet varieties were provided by All India Coordinated Research Project on Small Millets, University of Agricultural Sciences, Bengaluru, India. The cultivars of all the host species were grown in a polyhouse (temperature 25°C to 28°C) for 30 days in PVC pots containing red earth and fertilizers. The experiments were performed with three biological replicates and water control. The sporulation of *Magnaporthe* isolates was induced on oatmeal agar medium by growing the fungal cultures for 4 days in the dark conditions followed by 4 days in continuous light at 28°C and a spore suspension (1x10^5^ spores per mL) was prepared with 0.01% Tween 20 (CAS 9005-64-5, Fisher Scientific). The leaves were inoculated by punch inoculation method and the occurrence of disease was recorded after 5–7 days of inoculation.

### DNA isolation, sample preparation and whole genome sequencing

*Magnaporthe* isolates were grown in a sterilized liquid medium (Sucrose 1g, yeast extract 0.2g in 100mL of double distilled water) for three days in the dark condition at 200 RPM at 28°C. The mycelia were filtered and genomic DNA was extracted using a nucleo-pore gDNA fungal and bacterial mini kit (Genaxy, Cat.# NP-7006D). One microgram of genomic DNA was fragmented to obtain an average of 350bp fragments using Covaris (An instrument used for shearing DNA to desired size range). The paired-end libraries were prepared using a TruSeq DNA sample preparation kit (Cat. No. FC-121-2001, Illumina). The libraries were quantified using a bioanalyzer and quantitative PCR (qPCR). The clusters were generated using cBOT and paired-end sequencing was carried out with an Illumina HiSeq1000 instrument at Center for Cellular and Molecular Platforms (C-CAMP), Bengaluru, India.

### Whole genome assembly

The paired-end reads from Illumina were quality filtered using the FastX tool kit (version0.0.13.2). The paired reads with at least 80% of the bases having a quality score greater than Q30 (base accuracy of 99.9%) were chosen for further analysis. *De novo* genome assembly was performed using SOAPdenovo2 [7]. The whole genome assembly is available at NCBI/DDBJ/EMBL with the accession IDs; MG02 (LNTH000000000), MG03 (LNTJ000000000), MG04 (LNTK000000000), MG05 (LNTI000000000), MG07 (LNTL000000000), MG08 (LNTN000000000), MG10 (LNTM000000000), and MG12 (LNTO000000000). The genome described in this paper is version 1 for all the isolates. The raw sequence reads are deposited in NCBI SRA database under the accession number SRP067816.

### Pan-genome analysis

The pan-genome analysis was performed using Panseq tool [8] and the percentage of sequence identity cutoff was set to 90. Protein sequences were clustered using BLASTClust (identity = 100% and coverage = 100%).

### Gene prediction and annotation

The contigs of all isolates were used to predict genes using MAKER version 2.31.6 [9] by providing expressed sequences (ESTs, cDNA, and mRNA) of *Magnaporthe* retrieved from the National Centre for Biological Information (NCBI). The *ab initio* gene prediction was included in MAKER pipeline using *Magnaporthe* as a gene prediction model with Augustus. The protein domain structures and gene ontology (GO) terms were assigned using InterProScan 5 software [10]. Functional annotation of genes was done by searching homology against protein sequences of the Ascomycetes downloaded from a Uniprot database using BLASTP alignments with an e-value threshold of 1e-10.

### Analysis of repeat elements and transposon insertion sites

The genomic content of repetitive elements was analyzed using a RepeatMasker 4.0.2 tool (http://www.repeatmasker.org). The library of *Magnaporthe* repeat elements in Repbase was used as a reference library for repeat prediction. The copy number of repeat elements was analyzed using an in-house Perl script based on 70% coverage of each repeat element. Insertion sites of repeat elements, and the upstream and downstream flanking regions were identified using the Identification of Transposon Insertion Sites (ITIS) tool [11]. The supercontigs of *Magnaporthe oryzae* strain 70–15 were used as a reference to map paired reads of *Magnaporthe* isolates with an average mapping quality of 10. The fragment insert size in a library was 300bp and 10 mismatches were allowed while transposing a TE.

### Analysis of synonymous to non-synonymous substitution ratio (Ka/Ks)

The calculation of a synonymous (Ks) and non-synonymous (Ka) substitution rate was performed using a KaKs calculator [12]. This program calculates ka and ks using model selection and model averaging, implementing various approximate and maximum likelihood methods. We used an approximate Nei and Gojobori (NG) method [13] for estimating the Ka/Ks ratio of gene pairs.

### Analysis of gene families

The predicted genes for all the isolates were subjected to BLASTALL, followed by the clustering of orthologous genes by OrthoMCL-v2.0.9 [14]. The ortho groups with at least one gene from each *Magnaporthe* isolate were considered as core ortho groups (COGs). Among the COGs, the ortho groups with more than one copy of genes were considered as expanded gene families. To understand the overall representation of shared ortho groups among host-specific forms, we performed host-wise clustering (within rice, finger millet, foxtail millet, and buffel grass).

### Variant calling

For variant calling, high-quality Illumina reads with a PHRED score greater than 30 were mapped to the reference *Magnaporthe* genome 70–15 using BWA [15]. The variant calling was performed in parallel across all the *Magnaporthe* genomes using GATK according to GATK best practices recommendations [16] [17]. A phylogenetic tree from SNPs identified by GATK was constructed using the Archaeopteryx tool (https://sites.google.com/site/cmzmasek/home/software/archaeopteryx).

### Secretome analysis

Genes having ≤ 200 amino acids were selected for secretome analysis using SignalP-4.1[18]. Proteins with signal peptide were subjected to TMHMM 2.0c [19] and TargetP-1.1b [20] to remove proteins with transmembrane helices and proteins targeted to mitochondria, respectively. The filtered set of proteins was finally subjected for PredGPI to remove GPI-anchored proteins [21].

### Analyses of carbohydrate-active enzymes (CAZymes) in *Magnaporthe*

*Magnaporthe* protein sequences which have a signal peptide motif were subjected to CAZymes annotation using CAT [22] and dbCAN [23] web servers, which are based on the CAZy (Carbohydrate-Active Enzyme) database classification [24]. The results from both the servers were combined and the genes were classified based on CAZy database (http://www.cazy.org/Welcome-to-the-Carbohydrate-Active.html) like glycoside hydrolases (GHs), glycosyl transferases (GTs), polysaccharide lyases (PLs), carbohydrate esterases (CEs), carbohydrate-binding modules (CBMs) and auxillary activities (AAs) [24].

## Results

### Whole genome sequencing, assembly and functional annotation

**Fig 1:**
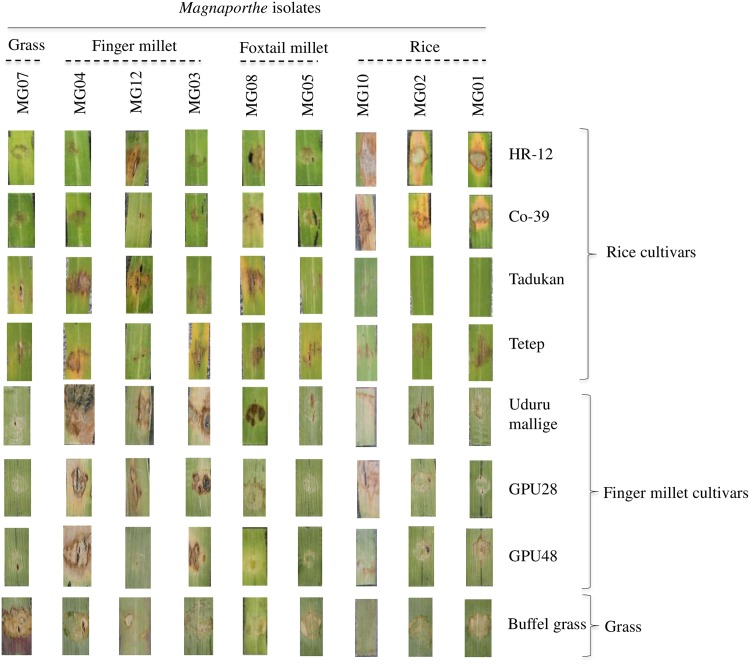
Cross-infection assay of rice and non-rice isolates. The spores of *Magnaporthe* isolates were artificially inoculated on various varieties of rice, finger millet and buffel grass.

The rice and non-rice *Magnaporthe* field isolates used in this study were isolated from infected leaf and neck tissues during the wet season of 2011 to 2014. The cross infection assay on various host species indicated the host-specific nature of each isolate as seen by the artificial inoculation method (Fig 1). All isolates used in this study did not show any cross infection. The rice isolates MG01, MG02 and MG10 produced disease lesions on susceptible rice varieties (HR-12 and Co-39). In case of finger millet isolates, MG04 was pathogenic to all the finger millet varieties. Whereas, MG03 and MG12 were pathogenic to Uduru mallige but non-pathogenic to GPU28 and GPU48. Similarly, the grass isolate, MG07 showed disease symptom on buffel grass. The high-quality Illumina paired-end reads of all isolates were assembled into scaffolds using the SOAPdenovo assembler version 2.04-r240 [25]. The assembly statistics for the genomes of all isolates is summarized in Table 1. The sequencing depth for all the genomes was greater than 40x.

**Table 1:** Assembly statistics and repeat content of rice and non-rice *Magnaporthe* isolates.

Host (Common Name)	Rice			Finger millet			Foxtail millet		*Buffel grass*
Host (Scientific Name)	*Oryza sativa*			*Eleusine coracana*			*Setaria italica*		*Cenchrus celiaris*
Isolate code	MG01*	MG10	MG02	MG03	MG12	MG04	MG05	MG08	MG07
Tissue	Leaf	Leaf	Neck	Leaf	Leaf	Neck	Leaf	Leaf	Leaf
Illumina PE reads (millions)	32	19	45	22	20	123	22	43	41
Sequence depth (X)	81	48	112	55	50	300	55	107	102
No. of scaffolds	17137	7336	19061	7872	8202	5972	5156	10135	12250
Largest scaffold length (nts)	186507	193896	388050	141810	106366	235178	124142	143558	110300
N50 (nts)	24781	27650	57789	25948	20755	51432	25058	25042	20306
Assembly size (Mb)	41	39	41	41	41	40	41	40	44
Number of genes	13494	13078	13448	13223	13081	12736	13235	13252	13252
Repeat content (%)	5.51	3.09	6.79	4.84	4.73	4.01	6.9	7.5	5.31

* Gowda et al. (2015)

The N50 ranged from 20–57 Kb across all isolates indicating good quality of the genome assemblies. The genome sizes of isolates ranged from 39 Mb to 43 Mb. The gene content of isolates was similar with gene numbers ranging from 12736 to 13494.

### *Magnaporthe* Pan-genome analysis

Knowledge of the genomic regions/genes specifically present in particular isolates can elaborate the mechanisms of host specificity. The whole genome comparison of MG07 with other rice (MG01, MG02, and MG10), finger millet (MG03, MG04, and MG12) and foxtail millet (MG05 and MG08) isolates showed that 1.40Mb, 1.03Mb, 1.9Mb genomic region was found to be unique in MG07, respectively. These genomic regions were identified with 152, 188, 416 protein-coding genes in MG07 as compared to rice, finger millet and foxtail millet isolates, respectively. Redundant genes were removed based on protein homology clustering by BLASTClust. Clustering resulted in a set of 549 non-redundant protein-coding genes. Out of which, 43 genes were exclusively present in MG07 but absent in other isolates (S1 Text). Most of these genes (32) were uncharacterized proteins and their functional relationship is yet to be deciphered. In addition to these, similar independent analysis between leaf and neck isolates of finger millet showed that 0.23Mb was uniquely present in MG04 neck isolate as compared to leaf isolates (MG03 and MG12), and this unique region was known to harbor 32 protein-coding genes. Few of these proteins encoded Avr-Pik (MG04_T12497-R1), isoflavone reductase (MG04_T03357-R1), ent-kaurene synthase (MG04_T00172-R1), initiation-specific alpha-1,6-mannosyltransferase (MG04_T04704-R1), isotrichodermin C-15 hydroxylase (MG04_T05900-R1), and aquaporin-2 (MG04_T09873-R1) proteins. Around 0.037Mb harboring 12 protein-coding genes (S2 Text) was found to be unique in MG02 neck isolate of rice as compared to rice leaf isolates MG01 and MG10. Among 12, only one gene encoded a short chain dehydrogenase/reductase (MG02_T12219-R1) and remaining proteins were uncharacterized.

### Distribution of transposable elements

**Fig 2:**
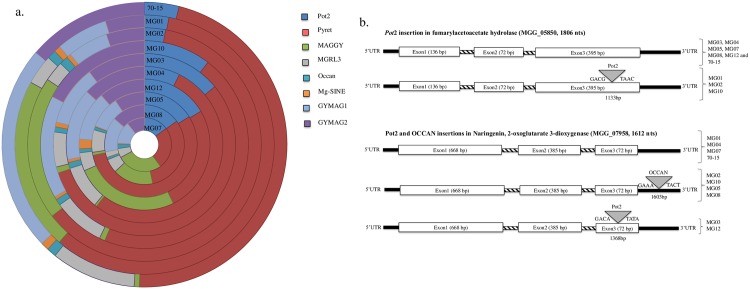
Distribution of transposable elements (a) and genic insertion of *Pot*2 and Occan (b) in rice and non-rice *Magnaporthe* isolates. Thick black bars indicate UTRs, and bars with crossed lines indicate Intron.

To compare the distribution of repeat elements among rice and non-rice isolates, whole genome assemblies were analyzed for repeat content. The overall repeat content ranged from ~3–7%, with the lowest in rice isolate MG10 (3.09%) and the highest in foxtail isolate MG08 (7.5%) (Table 1). The major proportion of repetitive DNA in the *Magnaporthe* genome consists of transposable elements (TEs). The important TEs are DNA transposon *Pot*2 and Occan [26] [27], LTR retro transposons Pyret [28], MAGGY [29], MGLR3 [30], Grasshopper [31] and SINE like element Mg-SINE [32]. Some of the TEs showed a significant copy number variation among different host-specific forms of *Magnaporthe* (Fig 2a; S1 Table). There were fewer than 80 copies of *Pot*2 in foxtail millet and buffel grass isolates; on the contrary, the Pyret element was highly enriched (more than 300 copies) in foxtail millet isolates.

Rice, finger millet and buffel grass isolates showed moderate copies of Pyret ranging from 81 to 172. Rice and foxtail millet isolates showed higher (96 to 242 copies) copy number of MAGGY, whereas fewer than 10 copies were present in finger millet isolates. Similarly, Mg-SINE was found in fewer copies in non-rice isolates as compared to rice isolates (except MG10). Few copies of *Grasshopper* (*grh*) were present in the finger millet subset of *Magnaporthe* population but, absent in rice, foxtail millet and buffel grass isolates. Other elements such as Occan, MGRL3, and GYMAG were distributed in variable proportions among rice and non-rice sub populations.

### Footprints of transposable elements in *Magnaporthe* genomes

The genome-wide insertion sites of transposable elements (TEs) were inferred using the ITIS tool [11]. The repeat insertion sites were predicted based on the 70–15 gene model as a reference. The copy number of each repeat element and the number of TE insertions are summarized in S2 Table. The number of insertions showed a variable trend for each repeat element. The overall rate of *Pot*2 insertion was higher in rice, followed by finger millet, foxtail millet and buffel grass isolates. The Occan and Pyret insertions were higher in rice and foxtail millet, followed by finger millet and buffel grass isolates. MAGGY and Fosbury insertions were absent in finger millet and buffel grass isolates. Interestingly, MGR583 insertions were higher in buffel grass followed by foxtail millet, rice and finger millet isolate MG12. A very sparse distribution of MGRL-3 was noticed across all isolates.

We particularly focused on TE insertions in genic regions considering their impact on gene functions. Some of the genic targets were conserved across the *Magnaporthe* isolates from the same host species for example, the fumarylacetoacetate hydrolase (MGG_05850) gene was mutated in all the rice isolates (Fig 2b). On the contrary, some genic insertions were unique to each isolate (S3 Table). Some genes were targeted by different transposable elements in different isolates; like the naringenin, 2-oxoglutarate 3-dioxygenase gene (MGG_07958) was disrupted by *Pot*2 in the MG03 and MG12 isolates whereas, in MG02, MG10, MG05, MG08 it was disrupted by Occan (Fig 2b). Additionally, genic insertion sites were conserved to some extent between isolates derived from the same host species (S3 Table). For instance, MG01 and MG02 shared more than 50% *Pot*2 insertions in genes such as dipeptidyl peptidase (MGG_05041), beta-fructofuranosidase (MGG_07853), MFS transporter (MGG_09130) and nitrate reductase (MGG_14232) (S3 Table). All the finger millet isolates did not show any common *Pot*2 footprints; however pairwise similarities of *Pot*2 insertions were observed between MG03-MG12, MG02-MG04.

### Genetic relatedness based on single nucleotide polymorphisms (SNPs)

**Fig 3:**
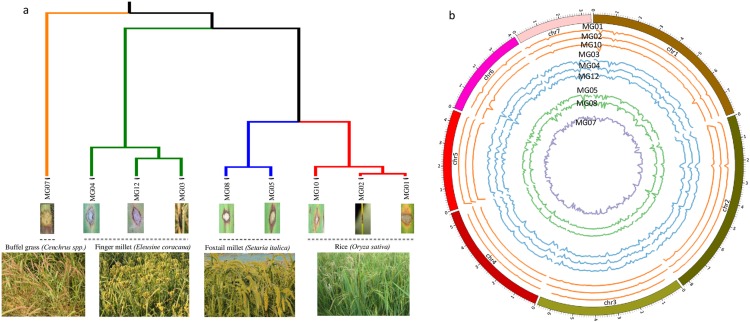
Genetic relatedness based on genome-wide SNPs of rice and non-rice isolates. (a) Chromosome-wise distribution of SNP density in rice and non-rice *Magnaporthe* isolates. The outer circle represents seven chromosomes of reference genome 70–15. The subsequent circles in inward order represent SNP density of rice isolates (MG01, MG02, and MG10), finger millet isolates (MG03, MG04, and MG12), foxtail millet isolates (MG05 and MG08) and buffel grass isolate MG07. (b) Genome-wide SNP based dendrogram was constructed based on UPGMA method.

Single nucleotide polymorphism (SNPs) is one of the important mechanisms generating variability across pathogen genomes. The multi-sample variant calling using GATK yielded a higher number of SNPs in finger millet and grass isolates. There were conserved peaks of SNPs within isolates from the same host species of *Magnaporthe* (Fig 3a).

The finger millet isolate MG04, showed the highest number of SNPs and INDELs followed by the buffel grass isolate MG07 (S4 Table). A genome-wide SNP based cladogram was generated to infer the genetic relatedness among the isolates (Fig 3b). The host-specific forms of the isolates were clustered in the same clade. The rice isolates were closely related to the foxtail millet isolates. The buffel grass (MG07) isolate was out grouped indicating a genetic dissimilarity with the other isolates. The genome-wide SNP density analysis showed the presence of 2 SNPs per 100 Kb in the rice isolates. The number of SNPs in the finger millet isolates ranged from 43 to 78 SNPs per 100 Kb. The foxtail millet isolates showed 13 and 14 SNPs per 100 Kb in MG05 and MG08, respectively. Similarly, the buffel grass isolate showed ~53 SNPs per 100 Kb of genomic region.

### Gene family duplications

**Fig 4:**
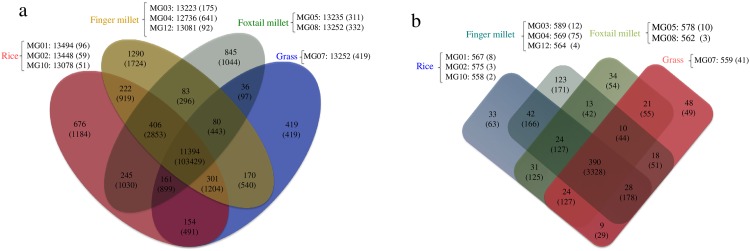
Host-wise clustering of proteomes (a) and secretome (b) of rice and non-rice *Magnaporthe* isolates. The number of gene families shared between the *Magnaporthe* species from different hosts (Rice, finger millet, foxtail millet, and Buffel grass) and a total number of clustered genes (numbers in parentheses) are indicated in Venn diagram. The numbers outside Venn diagram indicate, the isolate name from respective hosts, a total number of genes and singletons contributed by each isolate in parentheses.

The gene families were identified by comparing 118799 predicted proteins from all rice and non-rice *Magnaporthe* isolate genomes. A total of 9951 orthologous groups contained at least one gene from each *Magnaporthe* strain representing the core set of ortho groups. Since the main aim of this study was to compare the rice and non-rice isolates, we clustered the ortho groups based on the host species. The shared and unique ortho groups based on host-wise clustering (excluding 70–15 genes) are depicted in Fig 4a. There were 11394 common orthologous groups across all the host-specific form and 676, 1290, 845 and 419 unique ortho groups were seen in rice, finger millet, foxtail millet and buffel grass isolate, respectively. The finger millet neck isolate, MG04, (641 singletons) and the buffel grass isolate, MG07 (419 singletons) contributed the highest number of singletons to the respective host-specific clusters (Fig 4a). The majority of singletons were uncharacterized, hypothetical/predicted proteins. Interestingly, a set of 74 singletons was found to be similar to the uncharacterized proteins of *Magnaporthe poae* (strain ATCC 64411), a fungus from the *Magnaporthe* genus, which is associated with root infection [33]. The majority of genes (71 genes) sharing a similarity with *M*. *poae* belonged to the non-rice *Magnaporthe* isolates.

The orthologous groups having more than two copies of genes in the *Magnaporthe* isolate were considered as duplicated gene families. Some genes having globally important functions such as generation of nutrition, substrate transporters, genes involved in secondary metabolite pathways, and virulence were present in more than 5 copies in all the *Magnaporthe* strains including reference strain 70–15 (Table 2). For example, beta-glucosidase was present in more than 10 copies in all the isolates. Beta-glucosidase is important in generating energy in the form of glucose from cellooligosaccharides such as cellobiose, which is generated from the degradation of plant cellulose. Other important genes such as alpha-mannosidase, chitin synthase, glycerate kinase, ABC transporter CDR4 and multidrug resistance proteins were also present in multiple copies.

**Table 2:** Gene family expansions in rice and non-rice *Magnaporthe* isolates.

Host (Common Name)	Rice				Finger millet			Foxtail millet		Buffel grass
Host (Scientific Name)	*Oryza sativa*				*Eleusine coracana*			*Setaria italica*		*Cenchrus celiaris*
Gene annotation (gene length)	70–15	MG01	MG10	MG02	MG03	MG04	MG12	MG05	MG08	MG07
Hypothetical protein (1624 aa)	24	30	34	27	28	29	24	35	32	32
Beta-glucosidase 1 (569 aa)	11	11	11	11	12	13	12	11	11	10
Predicted protein (90 aa)	122	0	0	0	1	0	1	0	0	0
Glycerate kinase (488 aa)	12	8	11	9	10	11	12	10	10	9
Aspergillopepsin-F (431 aa)	9	9	9	9	9	10	9	9	9	7
Hypothetical protein (304 aa)	8	12	6	8	8	13	7	2	2	7
Glucan endo-1,3-alpha-glucosidase agn1 (432 aa)	9	7	7	7	7	8	6	7	7	7
Multidrug resistance protein 1 (1627 aa)	5	5	5	5	5	7	5	5	5	5
ABC transporter CDR4 (1621 aa)	5	5	5	5	6	5	5	5	5	5
Pre-mRNA-splicing factor ATP-dependent RNA helicase prp16 (1000 aa)	5	5	5	5	5	5	5	5	5	5
Predicted protein (808 aa)	5	3	5	3	7	6	5	7	5	4
Alpha-mannosidase (1101 aa)	5	5	5	4	5	5	4	5	5	5
Non-ribosomal peptide synthetase (2056 aa)	3	5	3	5	6	7	7	4	3	6
Calcium-transporting ATPase 1 (1287 aa)	3	4	4	4	4	5	4	4	4	5
Bifunctional P-450:NADPH-P450 reductase (1090 aa)	4	4	4	4	4	4	4	4	5	4

### Genes under positive selection

To know the genes under positive selection, we calculated the ratio of asynonymous to synonymous substitutions (Ka/Ks). The pairwise comparisons among the field isolates and with the reference *Magnaporthe* strain 70–15, yielded many genes under positive selection with a Ka/Ks ratio > 1 and with a probability value (Fischer exact test) < 0.05 (Table 3; S5 Table). The ratio of nucleotide substitutions (Ka/Ks) is used to analyze the function and evolution of coding regions in the genome. The comparison of isolates from the same host species did not show genes under positive selection except for a few genes among finger millet isolates. The candidate gene repertoire under positive selection encompassed of well-characterized genes involved in differentiation, virulence, male fertility, such as conidial yellow pigment biosynthesis polyketide synthase (PKS), clock-controlled pheromone ccg-4, serine/threonine protein kinase, medusa, feruloyl esterase B and many uncharacterized proteins under diversifying selection with a Ka/Ks ratio >1. In addition to these genes, many uncharacterized genes and hypothetical proteins were found to be under positive selection.

**Table 3:** Genes under positive selection.

Gene pair	Isolate pair	Functional Annotation	Ka/Ks	P-Value (Fisher)
MG01_T08864-MGG_08281T0	MG01vs 70–15	Conidial yellow pigment biosynthesis polyketide synthase	2.69031	0.0425223
MG02_T12663-MGG_17269T0	MG02 vs 70–15	Uncharacterised protein	9.08692	0.00573745
MG03_T11341-MG01_T12082	MG01 vs MG03	Protein kinase domain-containing protein	1.81954	0.0139821
MG04_T01496-MG01_T12653	MG04 vs MG01	Minor extracellular protease vpr	1.52119	0.000178351
MG10_T07073-MG04_T09244	MG04 vs MG10	Uncharacterised protein	2.75634	0.0316628
MG05_T07165-MG03_T10154	MG03 vs MG05	Uncharacterised protein	5.52728	0.0447517
MG05_T09521-MG03_T13084	MG03 vs MG05	Clock-controlled pheromone ccg-4	1.64328	0.0210628
MG05_T07165-MG03_T10154	MG03 vs MG05	Glucan 1,3-beta-glucosidase	5.52728	0.0447517
MG07_T11513-MG02_T12472	MG02 vs MG07	Medusa	8.51519	0.0310333
MG08_T03628-MG03_T01354	MG03 vs MG08	Uncharacterised protein	2.22945	0.0135478
MG04_T07733-MG01_T06649	MG01 vs MG04	Feruloyl esterase B	1.52957	0.0112177
MG12_T10806-MGG_13283T0	MG12 vs 70–15	Uncharacterised protein	2.64201	0.0367329

### Secretory proteins in *Magnaporthe* genomes

Candidate effectors are small protein entities, which assist in pathogenesis and enhance the infection process. We analyzed the proteomes of rice and non-rice *Magnaporthe* isolates to identify the putative candidate effector proteins. On an average, 4.3% of the total proteome encoded for secretory proteins, in all rice and non-rice isolates. To compare the repertoire of candidate effectors across all *Magnaporthe* isolate genomes, the predicted effectors were clustered using OrthoMCL. The host-wise clustering resulted in 390 common orthologous gene families between rice and non-rice isolates (Fig 4b). These common gene families encoded proteins with LysM, hydrophobin and CAP protein family (Pfam) domains, which are necessary for the growth and virulence of fungus (S6 Table).

Fifteen ortho groups of effectors were uniquely present in finger millet isolates, out of which one of the ortho group coded for pectate lyase B (The CAZymes analysis also indicated the presence of pectate lyase exclusively in finger millet isolates). Another set of 15 ortho groups of effectors was present in foxtail millet isolates exclusively. All gene family members of this foxtail millet specific groups were putative uncharacterized proteins. Interestingly, 32 gene families were present in all rice and non-rice isolates except, buffel grass isolate MG07. The majority of genes in these 32 gene families were also uncharacterized proteins with unknown functions, with the exception of one gene, which encoded the avirulence (*Avr*) gene *Avr-Pik*. In addition to this, 33, 123, 34 and 48 ortho groups were uniquely present in rice, finger millet, foxtail millet and buffel grass isolates, respectively. Among the isolate specific effectors, MG04 from finger millet showed the highest number of singletons (75), followed by the buffel grass isolate MG07 (41). The screening of MG04 singletons for putative pfam domains showed 23 proteins with known pfam domains with various functions involved in pathogenesis, such as the PTH11 integral membrane protein (CFEM domain), acid protease (Asp domain), acetylxylan esterase 2 (cutinase domain), GPI-anchored cell wall beta-1, 3-endoglucanase EglC (Glyco_hydro_76; CAZY) and 52 proteins with unknown functions. In case of buffel grass singletons, three proteins had zf-CCHC_3, zf-C2H2_jaz and HsbA pfam domains and 38 proteins had putative uncharacterized functions. Many *Avr* genes such as *Avr-Pik*, *Avr-Pii*, *Avr-Pita*, and host specificity factors *PWL2*, *PWL3* and *PWL4* were also present in the predicted secretome of rice and non-rice isolates. The PCR based validation of few Avr genes using gene-specific primers indicated variable distribution of *Avr* genes and host specificity factors in sequenced rice and non-rice *Magnaporthe* field isolates (S1 Fig). Only *Avr-Pizt* and *Avr-Pii* genes were majorly distributed among *Magnaporthe* isolates. However, the correlation of these effectors to host specificity can only be established by population scale analysis. Most of the candidate effectors involved in plant cell wall degradation and pathogenesis like pectin lyase, GPI-anchored cell wall beta-1, 3-endoglucanase, endoglucanase-4, hydrophobin-like protein MPG1 and cutinase were reported as secretory proteins involved in the *Magnaporthe* infection on rice [34]. In addition, five well-characterized proteins, namely MoCDIP1 to MoCDIP5 are known to induce cell death in rice. All rice and non-rice *Magnaporthe* isolates possessed these genes except rice isolate MG10, which lacked MoCDIP2 and MoCDIP3.

### Carbohydrate metabolism in *Magnaporthe*

**Fig 5:**
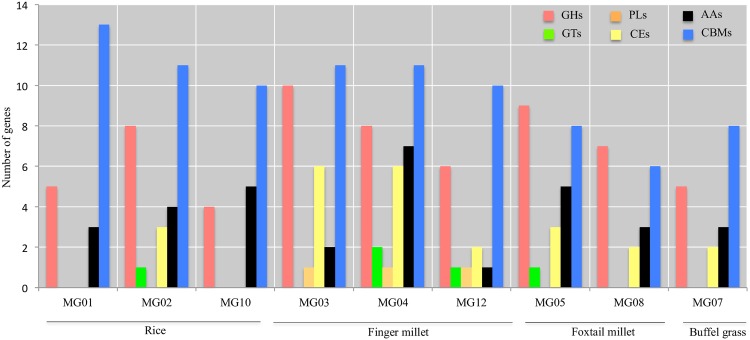
Distribution of secretory genes coding for carbohydrate-active enzymes in *Magnaporthe*.

Plant pathogens are known to produce various carbohydrate metabolism associated enzymes during invasive growth in plant tissues. The production of different classes of CAZymes plays an important role in the establishment of infection and the utilization of plant polysaccharides as an energy source. Thus, we screened the presence of CAZymes across predicted secretory proteins. Surprisingly, the distribution of CAZymes varied to some extent within isolates from the same host species (Fig 5).

Glycoside hydrolases (GHs), auxillary activities (AAs) and carbohydrate-binding modules (CBMs) were present in all isolates in variable proportions. Finger millet (MG04 and MG12) isolates had all classes of CAZymes. The carbohydrate esterases (CEs) were present in all isolates except in rice leaf isolates MG01 and MG10. Interestingly, polysaccharide lyases (PLs) were possessed only by finger millet isolates, MG03, MG04 and MG12 and absent in all the other isolates.

## Discussion

A comparison of recently isolated virulent rice, non-rice *Magnaporthe* field isolates was undertaken to address the elusive mechanisms of host specificity and the genomic factors involved therein. In addition, we also compared *Magnaporthe* isolates infecting leaf and neck tissue isolated from the same host cultivar. The genomes were analyzed in light of probable factors governing host specificity and genome plasticity.

### Genome size and gene content

Although the core genome size did not differ significantly across the *Magnaporthe* isolates, buffel grass isolate, MG07 showed a slightly higher genome size in comparison to other isolates. Thus, we performed pan-genome analysis to identify isolate specific genomic regions. The comparison revealed the genomic regions, which largely encoded uncharacterized proteins. Any increase in the genome size of pathogenic fungi can be driven by host adaptation followed by the lineage-specific expansion of virulence related genes and/or transposable elements [35]. A comparatively higher content of isolate specific effector proteins in MG07 might be the outcome of host adaptation. Based on an overall comparison, gene content variation seems to be one of the factors contributing to within species genetic variability in *Magnaporthe* lineages.

### Transposable elements

The recombination and movement of repetitive DNA elements in the genome are one of the main sources of variability in *Magnaporthe* [36]. The ubiquitous presence of almost all repeat elements in rice and non-rice *Magnaporthe* suggest that these elements existed prior to the evolution of host-specific *Magnaporthe* populations. The distribution of certain classes of repeat elements, such as Pyret and MAGGY, varied significantly across host-specific forms of *Magnaporthe*. Pyret and MAGGY were found to be the most active element in *Magnaporthe* genome instability upon stress induction [37]. Low copies of MAGGY were previously attributed to its comparatively recent acquisition in the genome by horizontal gene transfer [38]. However, the copy number of TEs might not directly correlate to their recent active status in the genome. Thus, we analyzed the genic insertion sites of these repeat elements in the genome. The insertions of TEs in genic regions can affect the virulence of fungus as exemplified by the *Pot*3 insertion in *Avr-Pita* leading to gain of virulence of *Magnaporthe* [39] [40]. An analysis of genic insertions among rice and non-rice isolates showed, *Pot*2 with the highest number of genic insertions (20 insertions) in all the isolates, except in the buffel grass isolate, where MGR583 showed higher genic copies (12 insertions) and only two *Pot*2 genic insertions. The insertion sites of TEs were conserved across the same host species (Fig 2b). Common genic targets and insertion sites within isolates from the same host species stipulate their common ancestral origin like the *Pot*2 insertion in the fumaryl acetoacetate gene in rice isolates (Fig 2b). The insertion of *Pot*2 in naringenin, 2-oxoglutarate 3-dioxygenase in the rice and foxtail millet isolates and in finger millet isolates indicates the variable activity of TEs in host-specific forms of *Magnaporthe*. The genic targets of TEs varied considerably across isolates (S3 Table) indicating their independent and sporadic evolution in the genome. Thus, the movement and activity of TEs in the genome might be subjective to the host, environmental stress factors and the evolutionary history of transposable elements, leading to the independent footprints of TEs in each field isolate of *Magnaporthe*. So far, most TE insertions and gain of virulence have been reported in *Avr* genes. The role of TEs in host specificity and the consequences of TE disruption in other genic regions of *Magnaporthe* are unexplored. The observed patterns of host specificity in TE insertions in this study will encourage the studies on non-avr genic disruptions by TEs.

### Effector proteins repertoire in host-specific forms of *Magnaporthe*

The secretome of *Magnaporthe* has been studied during the various stages of rice infection [34] [41] [42]. But the *Magnaporthe* effectors have not been studied at the genomic scale in non-rice host forms to understand their probable role as host specificity determinants. The effector proteins, which are shared between all the isolates, encoded the proteins with functional domains necessary for the growth and virulence of the fungus. The *Magnaporthe* protein containing a type III CVNH lectin with LysM domain insertion has a role in the early stages of plant infection [43]. The CAP1 is a cylase associated protein interacts with the Mac1 adenylate cylase gene in the cAMP signalling pathway, which is important in surface recognition and pathogenesis. The deletion mutants of CAP1 showed defects in growth and appressorium formation [44]. Similarly, hydrophobin mutants showed pleiotropic defects in growth, morphogenesis and virulence in *Magnaporthe*.

We observed a significant variation among rice and non-rice isolates with respect to effector proteins and CAZymes distribution. Particularly, finger millet isolate MG04 and buffel grass isolate MG07 showed the highest number of unique secretory proteins which might be specifically required to infect respective host species. Guyon and coworkers have demonstrated the differential expression of the candidate effectors in the fungal plant pathogen *Sclerotinia sclerotiorum* with a broad host range and showed the role of these effectors in host-specific interactions [45]. The isolate unique set of secretory proteins from our study can be explored further using the differential expressions to obtain pioneering insights into the role of effector proteins in host-specific and tissue-specific infections. We also analyzed the CAZyme subset of secretory protein since plant pathogenic fungi show large variations in their ability to degrade plant cell wall and the genes involved in carbohydrate metabolism [46]. Besides generating nutrition by degrading polysaccharides, CAZymes also have a role in virulence. We observed a distinct variation in the distribution of the CAZyme classes. PLs were specifically present in finger millet isolates. Finger millet isolates showed more classes of CAZymes unlike isolates of rice, foxtail millet and buffel grass. Neck isolates from rice and finger millet showed a similar CAZyme profile, except PLs, which was absent in the rice neck isolate, MG02. Overall, the CAZyme distribution followed a tissue and host-specific pattern indicating a specific requirement to degrade polysaccharides such as cellulose, hemicellulose, and pectins in particular tissues and hosts. One of the well-characterized cell wall degrading enzymes MoCDIP4, a putative endoglucanse, was shown to be responsible for inducing death in rice cells [34]. MoCDIP4 contained a glycosyl hydrolase family 61 domain and a fungal cellulose binding domain (CBD). The CBD acts as a pathogen associated molecular patterns (PAMP). Thus, the variability of secretory proteins, and especially, the enzymes involved in carbohydrate metabolism in rice and non-rice *Magnaporthe* needs to be explored to decipher the molecular mechanisms of pathogenesis and host-specific colonization.

### Nucleotide divergence

An analysis of nucleotide divergence in the pathogen population gives an important insight into pathogen evolution and speciation. We assessed the nucleotide diversity in terms of the Ka/Ks ratio and SNPs in rice and non-rice isolates. The Ka/Ks analysis did not show any genes under diversifying selection among rice isolates. However, the *Magnaporthe* isolates from finger millet neck and buffel grass showed a higher number of genetic loci under positive selection followed by the rest of the isolates from finger millet and foxtail millet. The PKS gene, which was under diversifying selection, was reported to show elevated expression during appressoria formation, and to have a role in the avirulence reaction along with ACE1 against the resistance gene *Pi33* [47]. The *ccg-4* is an essential pheromone in male fertility [48]. In the case of *Aspergillus nidulans*, medusa was shown to be involved in cell type modulation and the spatial organization of conidiophore structure. The deletion mutants showed defects in conidial differentiation and the formation of aberrant conidiophores [49]. The medusa protein in the case of *Magnaporthe* also might have a similar function. Another gene under positive selection was the SNARE protein, which has multiple functions including differentiation and it also affects virulence and the pathogenicity of fungus [50]. These genes and many other uncharacterized proteins under positive selection might be possible host specificity factors, involved in the maintenance of host boundaries or some putative candidates having a role in pathogenesis and survival. These genes can be a valuable resource for functional genomic studies to understand the evolution of host-specific forms of *Magnaporthe*.

The number of SNPs and SNP density per 100 Kb among rice isolates also was significantly lower. The overall low genetic diversity of rice isolates is correlated to asexual propagation and the uniform genetic background of its host. The higher genetic diversity of non-rice isolates could be due to genome plasticity and evolution in response to adaptation to new hosts and speciation [51] [52].

### Tissue specific infection

*Magnaporthe* isolates are able to infect different stages of plant growth, which includes leaf, collar, neck, node, and root. Whether the genetic makeup of *Magnaporthe* isolates infecting different host tissues is similar or distinct is an important question to be addressed. This information is necessary to design disease management strategies, especially for dual epidemics. This is the first genome-wide comparison between *Magnaporthe* isolates infecting different host tissues in rice and finger millet. Finger millet neck isolate MG04 showed presence of unique genomic regions, distinct variation in secretory proteins, CAZymes, high number of singletons and high nucleotide diversity. In comparison to finger millet neck isolate, the rice neck isolate (MG02) showed lesser variation as compared to the leaf counterparts MG01 and MG10. A recent study in rice has proposed that the more aggressive isolates from leaf epidemic can subsequently infect neck tissues and there is no tissue specialization in pathogen population [53]. This is partly supported by lesser variability exhibited by rice neck isolate MG02. However, the degree of genetic variability exhibited by finger millet neck isolate invokes the need for additional research in tissue-specific infection.

## Supporting Information

S1 Fig PCR based validation of avirulent genes in rice and non-rice *Magnaporthe* isolates. (DOCX)

S1 Table Distribution of repeat elements in rice and non-rice isolates. (DOCX)

S2 Table Copy number of the transposable element insertions in rice and non-rice isolates of *Magnaporthe*. (DOCX)

S3 Table Insertion sites of the transposable elements in genic regions of rice and non-rice isolates. (DOCX)

S4 Table SNPs and INDELs distribution across rice and non-rice *Magnaporthe* isolates. (DOCX)

S5 Table Genes under positive selection. (DOCX)

S6 Table Pfam domains of core set of candidate effectors gene families. (DOCX)

S1 Text Protein sequences of unique genes identified in MG07 compared to other *Magnaporthe* isolates. (DOCX)

S2 Text Protein sequences of unique genes identified in neck infecting *Magnaporthe* in fingermillet and rice. (DOCX)
