# Supplementary material for: Genome-Wide Comparison of Magnaporthe Species Reveals a Host-Specific Pattern of Secretory Proteins and Transposable Elements
Source: PLoS One. 2016 Sep 22;11(9):e0162458. doi: 10.1371/journal.pone.0162458 (PMC5033516; doi:10.1371/journal.pone.0162458)
Supplement: S1 Fig — (DOCX) [file pone.0162458.s001.docx]

**S1 Fig:** PCR based validation of avirulent genes in rice and non-rice *Magnaporthe* isolates

**
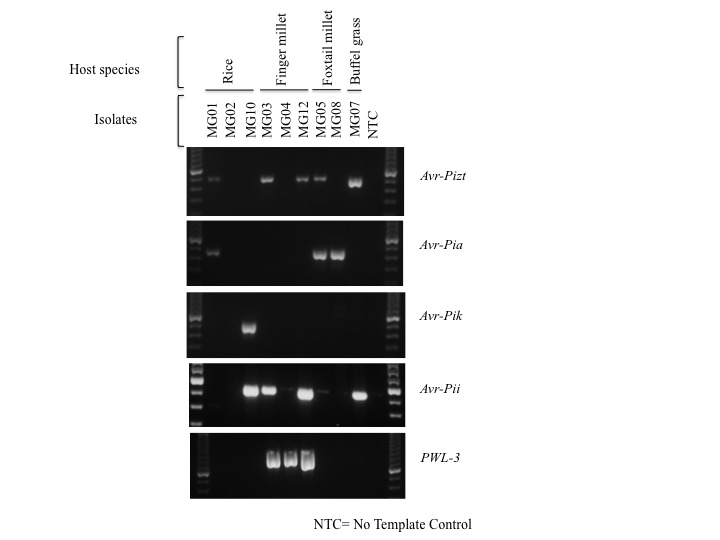
**

**Primer sequences used for PCR amplification**

| Gene | Product length (bp) | Forward Sequence | Reverse Sequence | Annealing Temparature | No. of PCR cycles |
| --- | --- | --- | --- | --- | --- |
| *PWL-3* | 834 | ACCTGCGAGTAAAAGCCTGA | TGTCTGCACCCCTCTCTCTT | 58 | 30 |
| *AVR-Pizt* | 493 | TCCCGTCACTTTCATTCTCC | CGAATTCCAGCCGAAGATAC | 58 | 30 |
| *AVR-Pia* | 450 | TGCACACAACAACCTCCATT | GGAATTTTCGGCAGAAATCA | 58 | 30 |
| *AVR-Pii* | 536 | ATTTATGCAGGCCCAAATCC | TGAAATTCCCGCAATAGTCC | 62 | 30 |
| *AVR-Pik* | 510 | ACTGCCACTCTGCACACATC | GTCAAACCTCCCTACGTTGC | 62 | 30 |
