# Supplementary material for: Genome-Wide Comparison of Magnaporthe Species Reveals a Host-Specific Pattern of Secretory Proteins and Transposable Elements
Source: PLoS One. 2016 Sep 22;11(9):e0162458. doi: 10.1371/journal.pone.0162458 (PMC5033516; doi:10.1371/journal.pone.0162458)
Supplement: S1 Table — (DOCX) [file pone.0162458.s002.docx]

**S1 Table:** Distribution of repeat elements in rice and non-rice isolates*.

| Host (Common Name) | Rice | | | | Finger millet | | | Foxtail millet | | *Buffel grass* |
| --- | --- | --- | --- | --- | --- | --- | --- | --- | --- | --- |
| Host (Scientific Name) | *Oryza sativa* | | | | *Eleusine coracana* | | | *Setaria italica* | | *Cenchrus celiaris* |
| Name of repeat element | 70-15 | MG01 | MG10 | MG02 | MG03 | MG12 | MG04 | MG05 | MG08 | MG07 |
| Pot2 | 278 | 325 | 191 | 441 | 105 | 130 | 96 | 72 | 71 | 47 |
| PYRET_LTR | 406 | 574 | 530 | 598 | 493 | 448 | 521 | 1198 | 1051 | 477 |
| PYRET_I-int | 67 | 113 | 129 | 103 | 95 | 121 | 172 | 323 | 311 | 81 |
| MAGGY_LTR | 345 | 164 | 8 | 242 | 7 | 8 | 6 | 125 | 96 | 6 |
| MAGGY_I-int | 166 | 194 | 3 | 446 | 0 | 3 | 0 | 366 | 407 | 1 |
| MGRL3_LTR | 93 | 110 | 80 | 112 | 107 | 95 | 109 | 69 | 81 | 111 |
| MGRL3_I-int | 11 | 11 | 2 | 8 | 3 | 5 | 10 | 1 | 6 | 4 |
| Occan_MG | 50 | 21 | 11 | 26 | 9 | 12 | 16 | 30 | 26 | 13 |
| Mg-SINE (U35230) | 10 | 51 | 3 | 65 | 9 | 6 | 8 | 15 | 19 | 11 |
| GYMAG1_I-int | 37 | 70 | 9 | 77 | 50 | 35 | 46 | 25 | 23 | 55 |
| GYMAG1_LTR | 214 | 238 | 109 | 248 | 153 | 164 | 148 | 114 | 128 | 225 |
| GYMAG2_I-int | 46 | 101 | 23 | 119 | 80 | 88 | 59 | 39 | 24 | 37 |
| GYMAG2_LTR | 157 | 196 | 87 | 187 | 131 | 128 | 113 | 92 | 90 | 128 |
| Grasshopper | 0 | 0 | 0 | 0 | 5 | 10 | 7 | 0 | 0 | 0 |

* The count is based on 70% coverage of each repeat element
