# Supplementary material for: Genome-Wide Comparison of Magnaporthe Species Reveals a Host-Specific Pattern of Secretory Proteins and Transposable Elements
Source: PLoS One. 2016 Sep 22;11(9):e0162458. doi: 10.1371/journal.pone.0162458 (PMC5033516; doi:10.1371/journal.pone.0162458)
Supplement: S2 Table — (DOCX) [file pone.0162458.s003.docx]

**S2 Table:** Copy number of the transposable element insertions in rice and non-rice isolates of *Magnaporthe*.

| **A. Rice isolates** | **MG01** | | **MG02** | | **MG10** | |
| --- | --- | --- | --- | --- | --- | --- |
| **Repeat name** | **No. of copies** | **No. of genic insertion (%)** | **No. of copies** | **No. of genic insertion** | **No. of copies** | **No. of genic insertion** |
| Fosbury | 30 | 4 | 27 | 4 | 19 | 1 |
| Grasshopper | 0 | 0 | 0 | 0 | 0 | 0 |
| MAGGY | 73 | 7 | 49 | 2 | 39 | 3 |
| MGR583 | 10 | 5 | 11 | 5 | 14 | 5 |
| MGRL3 | 1 | 0 | 0 | 0 | 0 | 0 |
| Occan | 37 | 1 | 32 | 4 | 31 | 3 |
| Pot2 | 196 | 20 | 182 | 16 | 182 | 20 |
| Pyret | 43 | 4 | 47 | 5 | 36 | 3 |

| **B. Finger millet isolates** | **MG03** | | **MG04** | | **MG12** | |
| --- | --- | --- | --- | --- | --- | --- |
| **Repeat name** | **No. of copies** | **No. of genic insertion** | **No. of copies** | **No. of genic insertion** | **No. of copies** | **No. of genic insertion** |
| Fosbury | 0 | 0 | 0 | 0 | 0 | 0 |
| Grasshopper | 0 | 0 | 0 | 0 | 0 | 0 |
| MAGGY | 0 | 0 | 0 | 0 | 0 | 0 |
| MGR583 | 0 | 0 | 0 | 0 | 14 | 9 |
| MGRL3 | 0 | 0 | 0 | 0 | 1 | 0 |
| Occan | 2 | 0 | 0 | 0 | 4 | 0 |
| Pot2 | 71 | 20 | 24 | 7 | 96 | 24 |
| Pyret | 4 | 0 | 0 | 0 | 3 | 0 |

| **C. Foxtail millet isolates** | **MG05** | | **MG08** | |
| --- | --- | --- | --- | --- |
| **Repeat name** | **No. of copies** | **No. of genic insertion** | **No. of copies** | **No. of genic insertion** |
| Fosbury | 17 | 1 | 18 | 0 |
| Grasshopper | 0 | 0 | 0 | 0 |
| MAGGY | 35 | 2 | 48 | 1 |
| MGR583 | 10 | 1 | 19 | 8 |
| MGRL3 | 0 | 0 | 0 | 0 |
| Occan | 14 | 2 | 13 | 2 |
| Pot2 | 40 | 5 | 43 | 6 |
| Pyret | 47 | 8 | 45 | 10 |

| **D. Buffel grass isolate** | **MG07** | |
| --- | --- | --- |
| **Repeat name** | **No. of copies** | **No. of genic insertion** |
| Fosbury | 0 | 0 |
| Grasshopper | 0 | 0 |
| MAGGY | 0 | 0 |
| MGR583 | 27 | 12 |
| MGRL3 | 0 | 0 |
| Occan | 3 | 1 |
| Pot2 | 3 | 2 |
| Pyret | 5 | 3 |
