# Supplementary material for: Genome-Wide Comparison of Magnaporthe Species Reveals a Host-Specific Pattern of Secretory Proteins and Transposable Elements
Source: PLoS One. 2016 Sep 22;11(9):e0162458. doi: 10.1371/journal.pone.0162458 (PMC5033516; doi:10.1371/journal.pone.0162458)
Supplement: S3 Table — (DOCX) [file pone.0162458.s004.docx]

**S3 Table:** Insertion sites of the transposable elements in genic regions of rice and non-rice isolates.

| Gene | Annotation (length) | Isolate | Repeat element |
| --- | --- | --- | --- |
| MGG_16563 | Predicted Protein (463 nt) | MG01 | Fosbury |
| MGG_18102 | Hypothetical Protein (219 nt) | MG01 | Fosbury |
| MGG_15948 | Predicted Protein (463 nt) | MG01 | Fosbury |
| MGG_17004 | Predicted Protein (463 nt) | MG01 | Fosbury |
| MGG_14570 | Hypothetical Protein (1560 nt) | MG01 | MAGGY |
| MGG_16563 | Predicted Protein (463 nt) | MG01 | MAGGY |
| MGG_18102 | Hypothetical Protein (219 nt) | MG01 | MAGGY |
| MGG_18113 | Hypothetical Protein (492 nt) | MG01 | MAGGY |
| MGG_09204 | Hypothetical Protein (1881 nt) | MG01 | MAGGY |
| MGG_16599 | Predicted Protein (463 nt) | MG01 | MAGGY |
| MGG_16984 | Hypothetical Protein (987 nt) | MG01 | MAGGY |
| MGG_13656 | Hypothetical Protein (2566 nt) | MG01 | MGR583 |
| MGG_13798 | Hypothetical Protein (2265 nt) | MG01 | MGR583 |
| MGG_14113 | Hypothetical Protein (839 nt) | MG01 | MGR583 |
| MGG_16395 | Atp-Dependent Zn Protease (2082 nt) | MG01 | MGR583 |
| MGG_12447 | Polyketide Synthase/Peptide Synthetase (12353 nt) | MG01 | MGR583 |
| MGG_16092 | Hypothetical Protein (457 nt) | MG01 | Occan |
| MGG_05041 | Dipeptidyl Peptidase Iii (2634 nt) | MG01 | Pot2 |
| MGG_05850 | Fumarylacetoacetate Hydrolase (1806 nt) | MG01 | Pot2 |
| MGG_07837 | Beta-Fructofuranosidase (2329 nt) | MG01 | Pot2 |
| MGG_09130 | Mfs Transporter (2906 nt) | MG01 | Pot2 |
| MGG_09420 | Hypothetical Protein (903 nt) | MG01 | Pot2 |
| MGG_10968 | Hypothetical Protein (1508 nt) | MG01 | Pot2 |
| MGG_14232 | Nitrate Reductase 1 (3113 nt) | MG01 | Pot2 |
| MGG_16081 | Hypothetical Protein (1964 nt) | MG01 | Pot2 |
| MGG_16561 | Hypothetical Protein (930 nt) | MG01 | Pot2 |
| MGG_16938 | Hypothetical Protein (965 nt) | MG01 | Pot2 |
| MGG_16960 | Hypothetical Protein (381 nt) | MG01 | Pot2 |
| MGG_17548 | Hypothetical Protein (741 nt) | MG01 | Pot2 |
| MGG_17640 | Hypothetical Protein (883 nt) | MG01 | Pot2 |
| MGG_18121 | Hypothetical Protein (1178 nt) | MG01 | Pot2 |
| MGG_00039 | Hypothetical Protein (3004 nt) | MG01 | Pot2 |
| MGG_02164 | Hypothetical Protein (579 nt) | MG01 | Pot2 |
| MGG_04254 | Hypothetical Protein (1040 nt) | MG01 | Pot2 |
| MGG_14589 | Hypothetical Protein (858 nt) | MG01 | Pot2 |
| MGG_15933 | Hypothetical Protein (3051 nt) | MG01 | Pot2 |
| MGG_18078 | Hypothetical Protein (308 nt) | MG01 | Pot2 |
| MGG_02218 | Hypothetical Protein (1005 nt) | MG01 | Pyret |
| MGG_15044 | Hypothetical Protein (250 nt) | MG01 | Pyret |
| MGG_17848 | Hypothetical Protein (1378 nt) | MG01 | Pyret |
| MGG_18074 | Hypothetical Protein (306 nt) | MG01 | Pyret |
| MGG_16563 | Predicted Protein (463 nt) | MG02 | Fosbury |
| MGG_16580 | Predicted Protein (691 nt) | MG02 | Fosbury |
| MGG_16885 | Predicted Protein (463 nt) | MG02 | Fosbury |
| MGG_17499 | Hypothetical Protein (1039 nt) | MG02 | Fosbury |
| MGG_16563 | Predicted Protein (463 nt) | MG02 | MAGGY |
| MGG_17424 | Hypothetical Protein (885 nt) | MG02 | MAGGY |
| MGG_13656 | Hypothetical Protein (2566 nt) | MG02 | MGR583 |
| MGG_13798 | Hypothetical Protein (2265 nt) | MG02 | MGR583 |
| MGG_13798 | Hypothetical Protein (2265 nt) | MG02 | MGR583 |
| MGG_14113 | Hypothetical Protein (839 nt) | MG02 | MGR583 |
| MGG_16395 | Atp-Dependent Zn Protease (2082 nt) | MG02 | MGR583 |
| MGG_07958 | Naringenin,2-Oxoglutarate 3-Dioxygenase (1612 nt) | MG02 | Occan |
| MGG_11529 | Hypothetical Protein (3345 nt) | MG02 | Occan |
| MGG_13058 | Hypothetical Protein (3068 nt) | MG02 | Occan |
| MGG_14664 | Hypothetical Protein (2552 nt) | MG02 | Occan |
| MGG_05041 | Dipeptidyl Peptidase Iii (2634 nt) | MG02 | Pot2 |
| MGG_05850 | Fumarylacetoacetate Hydrolase (1806 nt) | MG02 | Pot2 |
| MGG_07837 | Beta-Fructofuranosidase (2329 nt) | MG02 | Pot2 |
| MGG_09130 | Mfs Transporter (2906 nt) | MG02 | Pot2 |
| MGG_09420 | Hypothetical Protein (903 nt) | MG02 | Pot2 |
| MGG_14232 | Nitrate Reductase 1 (3113 nt) | MG02 | Pot2 |
| MGG_14967 | Tyrocidine Synthetase 1 (18383 nt) | MG02 | Pot2 |
| MGG_16081 | Hypothetical Protein (1964 nt) | MG02 | Pot2 |
| MGG_16938 | Hypothetical Protein (965 nt) | MG02 | Pot2 |
| MGG_17548 | Hypothetical Protein (741 nt) | MG02 | Pot2 |
| MGG_17640 | Hypothetical Protein (883 nt) | MG02 | Pot2 |
| MGG_18121 | Hypothetical Protein (1178 nt) | MG02 | Pot2 |
| MGG_02206 | Hypothetical Protein (1027 nt) | MG02 | Pot2 |
| MGG_15318 | Hypothetical Protein (1392 nt) | MG02 | Pot2 |
| MGG_16351 | Hypothetical Protein (440 nt) | MG02 | Pot2 |
| MGG_17758 | Hypothetical Protein (598 nt) | MG02 | Pot2 |
| MGG_02218 | Hypothetical Protein (1005 nt) | MG02 | Pyret |
| MGG_07386 | C6 Zinc Finger Domain-Containing Protein (3759 nt) | MG02 | Pyret |
| MGG_15044 | Hypothetical Protein (250 nt) | MG02 | Pyret |
| MGG_17848 | Hypothetical Protein (1378 nt) | MG02 | Pyret |
| MGG_18074 | Hypothetical Protein (306 nt) | MG02 | Pyret |
| MGG_00216 | Hypothetical Protein (493 nt) | MG03 | Pot2 |
| MGG_02330 | Hypothetical Protein (2294 nt) | MG03 | Pot2 |
| MGG_05035 | Hypothetical Protein (2053 nt) | MG03 | Pot2 |
| MGG_05554 | Hypothetical Protein (1645 nt) | MG03 | Pot2 |
| MGG_07958 | Naringenin,2-Oxoglutarate 3-Dioxygenase (1612 nt) | MG03 | Pot2 |
| MGG_09048 | Nacht And Ankyrin Domain-Containing Protein (3982 nt) | MG03 | Pot2 |
| MGG_14570 | Hypothetical Protein (1560 nt) | MG03 | Pot2 |
| MGG_14953 | Hypothetical Protein (2057 nt) | MG03 | Pot2 |
| MGG_15032 | Hypothetical Protein (1173 nt) | MG03 | Pot2 |
| MGG_16561 | Hypothetical Protein (930 nt) | MG03 | Pot2 |
| MGG_18109 | Hypothetical Protein (678 nt) | MG03 | Pot2 |
| MGG_02009 | Hypothetical Protein (3330 nt) | MG03 | Pot2 |
| MGG_02058 | Mitochondrial Chaperone Bcs1 (1909 nt) | MG03 | Pot2 |
| MGG_02290 | Hypothetical Protein (1472 nt) | MG03 | Pot2 |
| MGG_02793 | Glycosyl Hydrolase Family 76 Protein (3336 nt) | MG03 | Pot2 |
| MGG_07949 | Hypothetical Protein (2258 nt) | MG03 | Pot2 |
| MGG_08686 | Hypothetical Protein (2252 nt) | MG03 | Pot2 |
| MGG_10754 | Hypothetical Protein (1353 nt) | MG03 | Pot2 |
| MGG_16012 | Hypothetical Protein (785 nt) | MG03 | Pot2 |
| MGG_16051 | Hypothetical Protein (300 nt) | MG03 | Pot2 |
| MGG_02330 | Hypothetical Protein (2294 nt) | MG04 | Pot2 |
| MGG_14953 | Hypothetical Protein (2057 nt) | MG04 | Pot2 |
| MGG_02592 | Hypothetical Protein (3394 nt) | MG04 | Pot2 |
| MGG_06236 | Hypothetical Protein (1209 nt) | MG04 | Pot2 |
| MGG_09677 | Hypothetical Protein (1878 nt) | MG04 | Pot2 |
| MGG_10026 | Cystein Rich Protein (1098 nt) | MG04 | Pot2 |
| MGG_18122 | Hypothetical Protein (1784 nt) | MG04 | Pot2 |
| MGG_17645 | Hypothetical Protein (981 nt) | MG05 | Fosbury |
| MGG_15032 | Hypothetical Protein (1173 nt) | MG05 | MAGGY |
| MGG_17645 | Hypothetical Protein (981 nt) | MG05 | MAGGY |
| MGG_10716 | Hypothetical Protein (1162 nt) | MG05 | MGR583 |
| MGG_07958 | Naringenin,2-Oxoglutarate 3-Dioxygenase (1612 nt) | MG05 | Occan |
| MGG_17100 | Hypothetical Protein (680 nt) | MG05 | Occan |
| MGG_05501 | Hypothetical Protein (2470 nt) | MG05 | Pot2 |
| MGG_08398 | Hypothetical Protein (1533 nt) | MG05 | Pot2 |
| MGG_11928 | Hypothetical Protein (565 nt) | MG05 | Pot2 |
| MGG_16100 | Hypothetical Protein (790 nt) | MG05 | Pot2 |
| MGG_17258 | Hypothetical Protein (1437 nt) | MG05 | Pot2 |
| MGG_01949 | Ent-Kaurene Synthase (3192 nt) | MG05 | Pyret |
| MGG_08356 | Polysaccharide Deacetylase (2184 nt) | MG05 | Pyret |
| MGG_10434 | Hypothetical Protein (740 nt) | MG05 | Pyret |
| MGG_14593 | Hypothetical Protein (1904 nt) | MG05 | Pyret |
| MGG_15032 | Hypothetical Protein (1173 nt) | MG05 | Pyret |
| MGG_15145 | Hypothetical Protein (1287 nt) | MG05 | Pyret |
| MGG_15484 | Hypothetical Protein (451 nt) | MG05 | Pyret |
| MGG_14964 | Hypothetical Protein (429 nt) | MG05 | Pyret |
| MGG_00022 | Bassianolide Synthetase (12204 nt) | MG07 | MGR583 |
| MGG_00033 | Hypothetical Protein (1942 nt) | MG07 | MGR583 |
| MGG_00402 | Hypothetical Protein (1186 nt) | MG07 | MGR583 |
| MGG_01961 | Udp-Glucuronosyl/Udp-Glucosyltransferase (1532 nt) | MG07 | MGR583 |
| MGG_02350 | Hypothetical Protein (1201 nt) | MG07 | MGR583 |
| MGG_04206 | Hypothetical Protein (2613 nt) | MG07 | MGR583 |
| MGG_07985 | Hypothetical Protein (4555 nt) | MG07 | MGR583 |
| MGG_08725 | D-Lactate Dehydrogenase (4530 nt) | MG07 | MGR583 |
| MGG_09659 | S-(Hydroxymethyl)Glutathione Dehydrogenase (1316 nt) | MG07 | MGR583 |
| MGG_10407 | Hypothetical Protein (1793 nt) | MG07 | MGR583 |
| MGG_13974 | Hypothetical Protein (360 nt) | MG07 | MGR583 |
| MGG_14903 | Beta-Glucosidase 2 (4036 nt) | MG07 | MGR583 |
| MGG_16031 | Hypothetical Protein (401 nt) | MG07 | Occan |
| MGG_12049 | Hypothetical Protein (589 nt) | MG07 | Pot2 |
| MGG_16621 | Hypothetical Protein (684 nt) | MG07 | Pot2 |
| MGG_17258 | Hypothetical Protein (1437 nt) | MG07 | Pyret |
| MGG_16094 | Hypothetical Protein (2485 nt) | MG07 | Pyret |
| MGG_17562 | Hypothetical Protein (601 nt) | MG07 | Pyret |
| MGG_18113 | Hypothetical Protein (492 nt) | MG08 | MAGGY |
| MGG_10716 | Hypothetical Protein (1162 nt) | MG08 | MGR583 |
| MGG_00827 | Hypothetical Protein (1191 nt) | MG08 | MGR583 |
| MGG_05401 | Bifunctional P-450:Nadph-P450 Reductase (4175 nt) | MG08 | MGR583 |
| MGG_07919 | Hypothetical Protein (818 nt) | MG08 | MGR583 |
| MGG_08315 | 1-Phosphatidylinositol-4,5-Bisphosphate Phosphodiesterase Delta-1 (2249 nt) | MG08 | MGR583 |
| MGG_09602 | Hypothetical Protein (3216 nt) | MG08 | MGR583 |
| MGG_16113 | Hypothetical Protein (366 nt) | MG08 | MGR583 |
| MGG_16416 | Hypothetical Protein (686 nt) | MG08 | MGR583 |
| MGG_07958 | Naringenin,2-Oxoglutarate 3-Dioxygenase (1612 nt) | MG08 | Occan |
| MGG_17100 | Hypothetical Protein (680 nt) | MG08 | Occan |
| MGG_05501 | Hypothetical Protein (2470 nt) | MG08 | Pot2 |
| MGG_08398 | Hypothetical Protein (1533 nt) | MG08 | Pot2 |
| MGG_11928 | Hypothetical Protein (565 nt) | MG08 | Pot2 |
| MGG_16100 | Hypothetical Protein (790 nt) | MG08 | Pot2 |
| MGG_17258 | Hypothetical Protein (1437 nt) | MG08 | Pot2 |
| MGG_12203 | Hypothetical Protein (1766 nt) | MG08 | Pot2 |
| MGG_01949 | Ent-Kaurene Synthase (3192 nt) | MG08 | Pyret |
| MGG_08356 | Polysaccharide Deacetylase (2184 nt) | MG08 | Pyret |
| MGG_10434 | Hypothetical Protein (740 nt) | MG08 | Pyret |
| MGG_14593 | Hypothetical Protein (1904 nt) | MG08 | Pyret |
| MGG_15145 | Hypothetical Protein (1287 nt) | MG08 | Pyret |
| MGG_15484 | Hypothetical Protein (451 nt) | MG08 | Pyret |
| MGG_14982 | Hypothetical Protein (630 nt) | MG08 | Pyret |
| MGG_17584 | Hypothetical Protein (600 nt) | MG08 | Pyret |
| MGG_17799 | Hypothetical Protein (336 nt) | MG08 | Pyret |
| MGG_18065 | Hypothetical Protein (668 nt) | MG08 | Pyret |
| MGG_17285 | Hypothetical Protein (720 nt) | MG10 | Fosbury |
| MGG_17285 | Hypothetical Protein (720 nt) | MG10 | MAGGY |
| MGG_16407 | Predicted Protein (463 nt) | MG10 | MAGGY |
| MGG_16675 | Predicted Protein (463 nt) | MG10 | MAGGY |
| MGG_09427 | Hypothetical Protein (1797 nt) | MG10 | MGR583 |
| MGG_13591 | Mycocerosic Acid Synthase (7684 nt) | MG10 | MGR583 |
| MGG_16100 | Hypothetical Protein (790 nt) | MG10 | MGR583 |
| MGG_00010 | Hypothetical Protein (2308 nt) | MG10 | MGR583 |
| MGG_11005 | Hypothetical Protein (1653 nt) | MG10 | MGR583 |
| MGG_01835 | Inner Centromere Protein (5028 nt) | MG10 | Occan |
| MGG_07958 | Naringenin,2-Oxoglutarate 3-Dioxygenase (1612 nt) | MG10 | Occan |
| MGG_16756 | Hypothetical Protein (3370 nt) | MG10 | Occan |
| MGG_02207 | Hypothetical Protein (384 nt) | MG10 | Pot2 |
| MGG_02846 | Hypothetical Protein (5796 nt) | MG10 | Pot2 |
| MGG_05216 | Hypothetical Protein (3920 nt) | MG10 | Pot2 |
| MGG_05850 | Fumarylacetoacetate Hydrolase (1806 nt) | MG10 | Pot2 |
| MGG_05908 | Cytochrome P450 52A11 (1769 nt) | MG10 | Pot2 |
| MGG_07853 | Hypothetical Protein (1786 nt) | MG10 | Pot2 |
| MGG_08738 | Muts Protein (3823 nt) | MG10 | Pot2 |
| MGG_10236 | Hypothetical Protein (4568 nt) | MG10 | Pot2 |
| MGG_10456 | Hypothetical Protein (1144 nt) | MG10 | Pot2 |
| MGG_10537 | Hypothetical Protein (2207 nt) | MG10 | Pot2 |
| MGG_10912 | Mycocerosic Acid Synthase (8174 nt) | MG10 | Pot2 |
| MGG_10968 | Hypothetical Protein (1508 nt) | MG10 | Pot2 |
| MGG_14957 | Hypothetical Protein (2485 nt) | MG10 | Pot2 |
| MGG_15319 | Hypothetical Protein (531 nt) | MG10 | Pot2 |
| MGG_16486 | Hypothetical Protein (8150 nt) | MG10 | Pot2 |
| MGG_16611 | Hypothetical Protein (1152 nt) | MG10 | Pot2 |
| MGG_17298 | Predicted Protein (660 nt) | MG10 | Pot2 |
| MGG_18070 | Hypothetical Protein (662 nt) | MG10 | Pot2 |
| MGG_16952 | Hypothetical Protein (1209 nt) | MG10 | Pot2 |
| MGG_02218 | Hypothetical Protein (1005 nt) | MG10 | Pyret |
| MGG_07386 | C6 Zinc Finger Domain-Containing Protein (3759 nt) | MG10 | Pyret |
| MGG_16548 | Hypothetical Protein (1110 nt) | MG10 | Pyret |
| MGG_14967 | Tyrocidine Synthetase 1 (18383 nt) | MG12 | MGR583 |
| MGG_01906 | Nicotianamine Synthase 3 (1479 nt) | MG12 | MGR583 |
| MGG_02342 | Hypothetical Protein (2650 nt) | MG12 | MGR583 |
| MGG_08989 | Hypothetical Protein (1460 nt) | MG12 | MGR583 |
| MGG_09252 | Hypothetical Protein (570 nt) | MG12 | MGR583 |
| MGG_10436 | Hypothetical Protein (402 nt) | MG12 | MGR583 |
| MGG_12501 | Hypothetical Protein (1469 nt) | MG12 | MGR583 |
| MGG_13449 | Hypothetical Protein (2135 nt) | MG12 | MGR583 |
| MGG_13923 | Hypothetical Protein (582 nt) | MG12 | MGR583 |
| MGG_00216 | Hypothetical Protein (493 nt) | MG12 | Pot2 |
| MGG_05035 | Hypothetical Protein (2053 nt) | MG12 | Pot2 |
| MGG_05554 | Hypothetical Protein (1645 nt) | MG12 | Pot2 |
| MGG_07958 | Naringenin,2-Oxoglutarate 3-Dioxygenase (1612 nt) | MG12 | Pot2 |
| MGG_09048 | Nacht And Ankyrin Domain-Containing Protein (3982 nt) | MG12 | Pot2 |
| MGG_15145 | Hypothetical Protein (1287 nt) | MG12 | Pot2 |
| MGG_16486 | Hypothetical Protein (8150 nt) | MG12 | Pot2 |
| MGG_16960 | Hypothetical Protein (381 nt) | MG12 | Pot2 |
| MGG_18109 | Hypothetical Protein (678 nt) | MG12 | Pot2 |
| MGG_01856 | Hypothetical Protein (1764 nt) | MG12 | Pot2 |
| MGG_01944 | Hypothetical Protein (1339 nt) | MG12 | Pot2 |
| MGG_03426 | 3-Oxoacyl-[Acyl-Carrier-Protein] Reductase (895 nt) | MG12 | Pot2 |
| MGG_06807 | Hypothetical Protein (1428 nt) | MG12 | Pot2 |
| MGG_07571 | Lysm Domain-Containing Protein (1576 nt) | MG12 | Pot2 |
| MGG_08519 | Aflatoxin B1 Aldehyde Reductase Member 3 (1164 nt) | MG12 | Pot2 |
| MGG_10757 | Hypothetical Protein (1177 nt) | MG12 | Pot2 |
| MGG_11945 | Candidapepsin (2983 nt) | MG12 | Pot2 |
| MGG_15194 | Hypothetical Protein (723 nt) | MG12 | Pot2 |
| MGG_15393 | Hypothetical Protein (2141 nt) | MG12 | Pot2 |
| MGG_16077 | Hypothetical Protein (1018 nt) | MG12 | Pot2 |
| MGG_17237 | Hypothetical Protein (578 nt) | MG12 | Pot2 |
| MGG_17657 | Hypothetical Protein (700 nt) | MG12 | Pot2 |
| MGG_17746 | Hypothetical Protein (3125 nt) | MG12 | Pot2 |
| MGG_17747 | Hypothetical Protein (2207 nt) | MG12 | Pot2 |
