# Supplementary material for: Genome-Wide Comparison of Magnaporthe Species Reveals a Host-Specific Pattern of Secretory Proteins and Transposable Elements
Source: PLoS One. 2016 Sep 22;11(9):e0162458. doi: 10.1371/journal.pone.0162458 (PMC5033516; doi:10.1371/journal.pone.0162458)
Supplement: S4 Table — (DOCX) [file pone.0162458.s005.docx]

S4 Table. SNPs and INDELs distribution across rice and non-rice *Magnaporthe* isolates

| Host | Isolate | SNPs | INDELs | SNP density per 100 Kb | Host wise SNP density per 100 Kb |
| --- | --- | --- | --- | --- | --- |
| Rice | MG01 | 816 | 3552 | 2.1 | 2.41 |
| Rice | MG10 | 814 | 3235 | 3.1 |  |
| Rice | MG02 | 1248 | 3842 | 2.04 |  |
| Finger millet | MG03 | 17758 | 27484 | 43.5 | 53.63 |
| Finger millet | MG12 | 15702 | 26733 | 38.57 |  |
| Finger millet | MG04 | 32153 | 32448 | 78.83 |  |
| Foxtail millet | MG05 | 5359 | 13025 | 13.21 | 13.79 |
| Foxtail millet | MG08 | 5795 | 13358 | 14.36 |  |
| Buffel Grass | MG07 | 21541 | 31338 | 52.88 | 52.88 |
