# Supplementary material for: Genome-Wide Comparison of Magnaporthe Species Reveals a Host-Specific Pattern of Secretory Proteins and Transposable Elements
Source: PLoS One. 2016 Sep 22;11(9):e0162458. doi: 10.1371/journal.pone.0162458 (PMC5033516; doi:10.1371/journal.pone.0162458)
Supplement: S5 Table — (DOCX) [file pone.0162458.s006.docx]

**S5 Table:** Genes under positive selection.

| Gene IDs | Annotation | Ka/Ks ratio | P-value |
| --- | --- | --- | --- |
| MG01 to MG03 |  |  |  |
| MG03_T01354-MG01_T08151 | Similar to OOU_Y34scaffold01166g3 Uncharacterized protein (Magnaporthe oryzae (strain Y34)) | 2.22945 | 0.0135478 |
| MG03_T08781-MG01_T09529 | Similar to OOU_Y34scaffold00050g19 Uncharacterized protein (Magnaporthe oryzae (strain Y34)) | 2.31816 | 0.0476693 |
| MG03_T08853-MG01_T11615 | Similar to OOU_Y34scaffold00519g25 Uncharacterized protein (Magnaporthe oryzae (strain Y34)) | 1.68236 | 0.0234798 |
| MG03_T09555-MG01_T10712 | Similar to OOU_Y34scaffold00254g3 Uncharacterized protein (Magnaporthe oryzae (strain Y34)) | 4.10664 | 0.0487841 |
| MG03_T11341-MG01_T12082 | Similar to OOU_Y34scaffold00830g2 Protein kinase domain-containing protein (Magnaporthe oryzae (strain Y34)) | 1.81954 | 0.0139821 |
| MG03_T12452-MG01_T13254 | Similar to OOU_Y34scaffold01054g1 Ubiquinone/menaquinone biosynthesis methyltransferase (Magnaporthe oryzae (strain Y34)) | 1.52651 | 0.0498351 |
| MG01 to MG04 |  |  |  |
| MG04_T01496-MG01_T12653 | Similar to OOU_Y34scaffold00435g20 Minor extracellular protease vpr (Magnaporthe oryzae (strain Y34)) | 1.52119 | 0.000178351 |
| MG04_T07733-MG01_T06649 | Similar to MGG_09404 Feruloyl esterase B (Magnaporthe oryzae (strain 70-15 / ATCC MYA-4617 / FGSC 8958)) | 1.52957 | 0.0112177 |
| MG04_T09244-MG01_T05147 | Similar to MGG_16831 Uncharacterized protein (Magnaporthe oryzae (strain 70-15 / ATCC MYA-4617 / FGSC 8958)) | 3.27138 | 0.0163427 |
| MG04_T09248-MG01_T08151 | Similar to OOU_Y34scaffold01166g3 Uncharacterized protein (Magnaporthe oryzae (strain Y34)) | 2.01128 | 0.0286033 |
| MG04_T10150-MG01_T10712 | Similar to OOU_Y34scaffold00254g3 Uncharacterized protein (Magnaporthe oryzae (strain Y34)) | 4.10664 | 0.0487841 |
| MG04_T10752-MG01_T09165 | Similar to MGG_13283 Uncharacterized protein (Magnaporthe oryzae (strain 70-15 / ATCC MYA-4617 / FGSC 8958)) | 3.33282 | 0.0193922 |
| MG04_T11287-MG01_T11615 | Similar to OOU_Y34scaffold00519g25 Uncharacterized protein (Magnaporthe oryzae (strain Y34)) | 1.77492 | 0.00638019 |
| MG01 to MG05 |  |  |  |
| MG05_T09521-MG01_T10496 | Similar to OOU_Y34scaffold00287g12 Clock-controlled pheromone ccg-4 (Magnaporthe oryzae (strain Y34)) | 1.66938 | 0.0243989 |
| MG05_T11871-MG01_T11615 | Similar to MGG_10001 Uncharacterized protein (Magnaporthe oryzae (strain 70-15 / ATCC MYA-4617 / FGSC 8958)) | 1.68659 | 0.0112608 |
| MG01 to MG07 |  |  |  |
| MG07_T07472-MG01_T13127 | Similar to OOU_Y34scaffold00220g1 Uncharacterized protein (Magnaporthe oryzae (strain Y34)) | 2.86113 | 0.0249987 |
| MG07_T10550-MG01_T05693 | Similar to OOU_Y34scaffold00435g14 Uncharacterized protein (Magnaporthe oryzae (strain Y34)) | 3.99974 | 0.0400622 |
| MG07_T11513-MG01_T07632 | Similar to MGG_15985 Uncharacterized protein (Magnaporthe oryzae (strain 70-15 / ATCC MYA-4617 / FGSC 8958)) | 8.51519 | 0.0310333 |
| MG07_T11989-MG01_T11884 | Similar to MGCH7_ch7g68 Uncharacterized protein (Magnaporthe oryzae (strain 70-15 / ATCC MYA-4617 / FGSC 8958)) | 13.9094 | 0.00500448 |
| MG07_T12380-MG01_T10969 | Similar to OOU_Y34scaffold01027g1 Uncharacterized protein (Magnaporthe oryzae (strain Y34)) | 9.15927 | 0.0497728 |
| MG07_T12833-MG01_T13179 | Similar to OOW_P131scaffold01323g1 Uncharacterized protein (Magnaporthe oryzae (strain P131)) | 3.1251 | 0.00801844 |
| MG01 to MG08 |  |  |  |
| MG08_T11585-MG01_T11615 | Similar to OOU_Y34scaffold00519g25 Uncharacterized protein (Magnaporthe oryzae (strain Y34)) | 1.78355 | 0.0049872 |
| MG01 to MG12 |  |  |  |
| MG12_T03538-MG01_T09529 | Similar to OOU_Y34scaffold00050g19 Uncharacterized protein (Magnaporthe oryzae (strain Y34)) | 2.31816 | 0.0476693 |
| MG12_T10582-MG01_T10712 | Similar to OOU_Y34scaffold00254g3 Uncharacterized protein (Magnaporthe oryzae (strain Y34)) | 4.10664 | 0.0487841 |
| MG12_T10806-MG01_T09165 | Similar to MGG_13283 Uncharacterized protein (Magnaporthe oryzae (strain 70-15 / ATCC MYA-4617 / FGSC 8958)) | 2.64201 | 0.0367329 |
| MG12_T11764-MG01_T12082 | Similar to OOU_Y34scaffold00830g2 Protein kinase domain-containing protein (Magnaporthe oryzae (strain Y34)) | 1.81954 | 0.0139821 |
| MG01 to 70-15 |  |  |  |
| MG01_6660-MGG_16743T0 | Similar to MGG_16743 Hypothetical protein (Magnaporthe oryzae (strain 70-15 / ATCC MYA-4617 / FGSC 8958)) | 13.3745 | 0.0409685 |
| MG02 to MG03 |  |  |  |
| MG03_T01354-MG02_T10553 | Similar to OOU_Y34scaffold01166g3 Uncharacterized protein (Magnaporthe oryzae (strain Y34)) | 2.22945 | 0.0135478 |
| MG03_T08781-MG02_T09957 | Similar to OOU_Y34scaffold00050g19 Uncharacterized protein (Magnaporthe oryzae (strain Y34)) | 2.31816 | 0.0476693 |
| MG03_T08853-MG02_T11788 | Similar to OOU_Y34scaffold00519g25 Uncharacterized protein (Magnaporthe oryzae (strain Y34)) | 1.68236 | 0.0234798 |
| MG03_T09555-MG02_T10979 | Similar to OOU_Y34scaffold00254g3 Uncharacterized protein (Magnaporthe oryzae (strain Y34)) | 4.10664 | 0.0487841 |
| MG03_T11341-MG02_T12205 | Similar to OOU_Y34scaffold00830g2 Protein kinase domain-containing protein (Magnaporthe oryzae (strain Y34)) | 1.81954 | 0.0139821 |
| MG02 to MG04 |  |  |  |
| MG04_T09244-MG02_T05910 | Similar to MGG_16831 Uncharacterized protein (Magnaporthe oryzae (strain 70-15 / ATCC MYA-4617 / FGSC 8958)) | 3.27138 | 0.0163427 |
| MG04_T09248-MG02_T10553 | Similar to OOU_Y34scaffold01166g3 Uncharacterized protein (Magnaporthe oryzae (strain Y34)) | 2.22945 | 0.0135478 |
| MG04_T10150-MG02_T10979 | Similar to OOU_Y34scaffold00254g3 Uncharacterized protein (Magnaporthe oryzae (strain Y34)) | 4.10664 | 0.0487841 |
| MG04_T11287-MG02_T11788 | Similar to OOU_Y34scaffold00519g25 Uncharacterized protein (Magnaporthe oryzae (strain Y34)) | 1.62511 | 0.0215545 |
| MG02 to MG05 |  |  |  |
| MG05_T11871-MG02_T11788 | Similar to MGG_10001 Uncharacterized protein (Magnaporthe oryzae (strain 70-15 / ATCC MYA-4617 / FGSC 8958)) | 1.68659 | 0.0112608 |
| MG02 to MG07 |  |  |  |
| MG07_T06838-MG02_T10743 | Similar to OOU_Y34scaffold00308g38 Adhesin protein Mad1 (Magnaporthe oryzae (strain Y34)) | 1.35289 | 0.0392332 |
| MG07_T07472-MG02_T13092 | Similar to OOU_Y34scaffold00220g1 Uncharacterized protein (Magnaporthe oryzae (strain Y34)) | 2.95476 | 0.0435223 |
| MG07_T10550-MG02_T06841 | Similar to OOU_Y34scaffold00435g14 Uncharacterized protein (Magnaporthe oryzae (strain Y34)) | 3.99974 | 0.0400622 |
| MG07_T11513-MG02_T12472 | Similar to MGG_15985 Uncharacterized protein (Magnaporthe oryzae (strain 70-15 / ATCC MYA-4617 / FGSC 8958)) | 8.51519 | 0.0310333 |
| MG07_T12380-MG02_T11194 | Similar to OOU_Y34scaffold01027g1 Uncharacterized protein (Magnaporthe oryzae (strain Y34)) | 9.15927 | 0.0497728 |
| MG07_T12833-MG02_T13147 | Similar to OOW_P131scaffold01323g1 Uncharacterized protein (Magnaporthe oryzae (strain P131)) | 3.1251 | 0.00801844 |
| MG02 to MG08 |  |  |  |
| MG08_T11585-MG02_T11788 | Similar to OOU_Y34scaffold00519g25 Uncharacterized protein (Magnaporthe oryzae (strain Y34)) | 1.79883 | 0.00508838 |
| MG02 to MG12 |  |  |  |
| MG12_T03538-MG02_T09957 | Similar to OOU_Y34scaffold00050g19 Uncharacterized protein (Magnaporthe oryzae (strain Y34)) | 2.31816 | 0.0476693 |
| MG12_T10582-MG02_T10979 | Similar to OOU_Y34scaffold00254g3 Uncharacterized protein (Magnaporthe oryzae (strain Y34)) | 4.10664 | 0.0487841 |
| MG12_T10806-MG02_T09666 | Similar to MGG_13283 Uncharacterized protein (Magnaporthe oryzae (strain 70-15 / ATCC MYA-4617 / FGSC 8958)) | 2.64201 | 0.0367329 |
| MG12_T11764-MG02_T12205 | Similar to OOU_Y34scaffold00830g2 Protein kinase domain-containing protein (Magnaporthe oryzae (strain Y34)) | 1.81954 | 0.0139821 |
| MG02 to 70-15 |  |  |  |
| MG02_T09439-MGG_08281T0 | Similar to OOU_Y34scaffold00555g1 Conidial yellow pigment biosynthesis polyketide synthase (Fragment) (Magnaporthe oryzae (strain Y34)) | 2.68999 | 0.042516 |
| MG02_T12663-MGG_17269T0 | Similar to MGG_16179 Uncharacterized protein (Magnaporthe oryzae (strain 70-15 / ATCC MYA-4617 / FGSC 8958)) | 9.08692 | 0.00573745 |
| MG03 to MG04 |  |  |  |
| MG04_T08241-MG03_T11341 | Similar to MGG_14499 Serine/threonine protein kinase (Magnaporthe oryzae (strain 70-15 / ATCC MYA-4617 / FGSC 8958)) | 2.20033 | 0.0175526 |
| MG03 to MG05 |  |  |  |
| MG05_T00399-MG03_T01354 | Similar to OOU_Y34scaffold01166g3 Uncharacterized protein (Magnaporthe oryzae (strain Y34)) | 2.22945 | 0.0135478 |
| MG05_T05807-MG03_T08937 | Similar to OOU_Y34scaffold00694g3 Uncharacterized protein (Magnaporthe oryzae (strain Y34)) | 6.29854 | 0.0347445 |
| MG05_T07165-MG03_T10154 | Similar to MGG_16973 Uncharacterized protein (Magnaporthe oryzae (strain 70-15 / ATCC MYA-4617 / FGSC 8958)) | 5.52728 | 0.0447517 |
| MG05_T09503-MG03_T08781 | Similar to OOU_Y34scaffold00050g19 Uncharacterized protein (Magnaporthe oryzae (strain Y34)) | 2.31816 | 0.0476693 |
| MG05_T09521-MG03_T13084 | Similar to OOU_Y34scaffold00287g12 Clock-controlled pheromone ccg-4 (Magnaporthe oryzae (strain Y34)) | 1.64328 | 0.0210628 |
| MG05_T11041-MG03_T11341 | Similar to OOU_Y34scaffold00830g2 Protein kinase domain-containing protein (Magnaporthe oryzae (strain Y34)) | 1.82065 | 0.0140025 |
| MG03 to MG07 |  |  |  |
| MG07_T07472-MG03_T07453 | Similar to OOU_Y34scaffold00220g1 Uncharacterized protein (Magnaporthe oryzae (strain Y34)) | 2.03881 | 0.0348794 |
| MG07_T07835-MG03_T08853 | Similar to OOU_Y34scaffold00519g25 Uncharacterized protein (Magnaporthe oryzae (strain Y34)) | 1.6426 | 0.0317928 |
| MG07_T08124-MG03_T10674 | Similar to OOU_Y34scaffold00665g21 Uncharacterized protein (Magnaporthe oryzae (strain Y34)) | 11.2494 | 0.0137724 |
| MG07_T08875-MG03_T10966 | Similar to OOU_Y34scaffold00652g3 Uncharacterized protein (Magnaporthe oryzae (strain Y34)) | 9.18501 | 0.0365299 |
| MG07_T11161-MG03_T13033 | Similar to OOU_Y34scaffold00517g3 Uncharacterized protein (Magnaporthe oryzae (strain Y34)) | 6.23405 | 0.00652638 |
| MG03 to MG08 |  |  |  |
| MG08_T03628-MG03_T01354 | Similar to OOU_Y34scaffold01166g3 Uncharacterized protein (Magnaporthe oryzae (strain Y34)) | 2.22945 | 0.0135478 |
| MG08_T06466-MG03_T08937 | Similar to OOU_Y34scaffold00694g3 Uncharacterized protein (Magnaporthe oryzae (strain Y34)) | 6.29854 | 0.0347445 |
| MG08_T09919-MG03_T08781 | Similar to OOU_Y34scaffold00050g19 Uncharacterized protein (Magnaporthe oryzae (strain Y34)) | 2.31816 | 0.0476693 |
| MG08_T11289-MG03_T11341 | Similar to OOU_Y34scaffold00830g2 Protein kinase domain-containing protein (Magnaporthe oryzae (strain Y34)) | 1.81954 | 0.0139821 |
| MG03 to MG10 |  |  |  |
| MG10_T10527-MG03_T08781 | Similar to OOU_Y34scaffold00050g19 Uncharacterized protein (Magnaporthe oryzae (strain Y34)) | 2.31816 | 0.0476693 |
| MG10_T11272-MG03_T01354 | Similar to OOU_Y34scaffold01166g3 Uncharacterized protein (Magnaporthe oryzae (strain Y34)) | 2.22945 | 0.0135478 |
| MG10_T12105-MG03_T11341 | Similar to OOU_Y34scaffold00830g2 Protein kinase domain-containing protein (Magnaporthe oryzae (strain Y34)) | 1.81954 | 0.0139821 |
| MG10_T12454-MG03_T09555 | Similar to OOU_Y34scaffold00254g3 Uncharacterized protein (Magnaporthe oryzae (strain Y34)) | 4.10664 | 0.0487841 |
| MG03 to MG12 |  |  |  |
| MG12_T02367-MG03_T01354 | Similar to OOU_Y34scaffold01166g3 Uncharacterized protein (Magnaporthe oryzae (strain Y34)) | 2.22945 | 0.0135478 |
| MG03 to 70-15 |  |  |  |
| MG03_T01354-MGG_05393T0 | Similar to OOU_Y34scaffold01166g3 Uncharacterized protein (Magnaporthe oryzae (strain Y34)) | 2.22945 | 0.0135478 |
| MG03_T08781-MGG_16930T0 | Similar to OOU_Y34scaffold00050g19 Uncharacterized protein (Magnaporthe oryzae (strain Y34)) | 2.31816 | 0.0476693 |
| MG03_T08853-MGG_10001T0 | Similar to OOU_Y34scaffold00519g25 Uncharacterized protein (Magnaporthe oryzae (strain Y34)) | 1.68236 | 0.0234798 |
| MG03_T09555-MGG_17266T0 | Similar to OOU_Y34scaffold00254g3 Uncharacterized protein (Magnaporthe oryzae (strain Y34)) | 4.10664 | 0.0487841 |
| MG04 to MG05 |  |  |  |
| MG05_T00399-MG04_T09248 | Similar to OOU_Y34scaffold01166g3 Uncharacterized protein (Magnaporthe oryzae (strain Y34)) | 2.22945 | 0.0135478 |
| MG04 to MG07 |  |  |  |
| MG07_T06355-MG04_T09244 | Similar to OOU_Y34scaffold00071g70 Uncharacterized protein (Magnaporthe oryzae (strain Y34)) | 3.16804 | 0.0244737 |
| MG07_T07472-MG04_T10342 | Similar to OOU_Y34scaffold00220g1 Uncharacterized protein (Magnaporthe oryzae (strain Y34)) | 2.03881 | 0.0348794 |
| MG07_T07835-MG04_T11287 | Similar to OOU_Y34scaffold00519g25 Uncharacterized protein (Magnaporthe oryzae (strain Y34)) | 1.61092 | 0.0199312 |
| MG07_T07838-MG04_T11707 | Similar to OOU_Y34scaffold00694g3 Uncharacterized protein (Magnaporthe oryzae (strain Y34)) | 6.91186 | 0.0222539 |
| MG07_T08124-MG04_T06438 | Similar to OOU_Y34scaffold00665g21 Uncharacterized protein (Magnaporthe oryzae (strain Y34)) | 11.2494 | 0.0137724 |
| MG07_T08875-MG04_T05561 | Similar to OOU_Y34scaffold00652g3 Uncharacterized protein (Magnaporthe oryzae (strain Y34)) | 9.18501 | 0.0365299 |
| MG07_T11161-MG04_T10224 | Similar to OOU_Y34scaffold00517g3 Uncharacterized protein (Magnaporthe oryzae (strain Y34)) | 6.23405 | 0.00652638 |
| MG07_T12980-MG04_T08595 | Similar to MGG_17256 Uncharacterized protein (Magnaporthe oryzae (strain 70-15 / ATCC MYA-4617 / FGSC 8958)) | 1.89508 | 0.0427592 |
| MG04 to MG08 |  |  |  |
| MG08_T03628-MG04_T09248 | Similar to OOU_Y34scaffold01166g3 Uncharacterized protein (Magnaporthe oryzae (strain Y34)) | 2.22945 | 0.0135478 |
| MG04 to MG10 |  |  |  |
| MG10_T07073-MG04_T09244 | Similar to OOU_Y34scaffold00071g70 Uncharacterized protein (Magnaporthe oryzae (strain Y34)) | 2.75634 | 0.0316628 |
| MG10_T11272-MG04_T09248 | Similar to OOU_Y34scaffold01166g3 Uncharacterized protein (Magnaporthe oryzae (strain Y34)) | 2.22945 | 0.0135478 |
| MG10_T12454-MG04_T10150 | Similar to OOU_Y34scaffold00254g3 Uncharacterized protein (Magnaporthe oryzae (strain Y34)) | 4.10664 | 0.0487841 |
| MG04 to MG12 |  |  |  |
| MG12_T02367-MG04_T09248 | Similar to OOU_Y34scaffold01166g3 Uncharacterized protein (Magnaporthe oryzae (strain Y34)) | 2.22945 | 0.0135478 |
| MG12_T11764-MG04_T08241 | Similar to OOU_Y34scaffold00830g2 Protein kinase domain-containing protein (Magnaporthe oryzae (strain Y34)) | 2.20033 | 0.0175526 |
| MG04 to 70-15 |  |  |  |
| MG04_T09244-MGG_16831T0 | Similar to MGG_16831 Uncharacterized protein (Magnaporthe oryzae (strain 70-15 / ATCC MYA-4617 / FGSC 8958)) | 3.27138 | 0.0163427 |
| MG04_T09248-MGG_05393T0 | Similar to OOU_Y34scaffold01166g3 Uncharacterized protein (Magnaporthe oryzae (strain Y34)) | 2.22945 | 0.0135478 |
| MG04_T10150-MGG_17266T0 | Similar to OOU_Y34scaffold00254g3 Uncharacterized protein (Magnaporthe oryzae (strain Y34)) | 4.10664 | 0.0487841 |
| MG04_T11287-MGG_10001T0 | Similar to OOU_Y34scaffold00519g25 Uncharacterized protein (Magnaporthe oryzae (strain Y34)) | 1.62511 | 0.0215545 |
| MG05 to MG07 |  |  |  |
| MG07_T07835-MG05_T11871 | Similar to OOU_Y34scaffold00519g25 Uncharacterized protein (Magnaporthe oryzae (strain Y34)) | 1.58811 | 0.0267075 |
| MG07_T08696-MG05_T03867 | Similar to MGG_16951 Uncharacterized protein (Magnaporthe oryzae (strain 70-15 / ATCC MYA-4617 / FGSC 8958)) | 2.9896 | 0.0361424 |
| MG07_T11989-MG05_T12609 | Similar to MGCH7_ch7g68 Uncharacterized protein (Magnaporthe oryzae (strain 70-15 / ATCC MYA-4617 / FGSC 8958)) | 13.9094 | 0.00500448 |
| MG07_T12083-MG05_T11995 | Similar to MGG_04195 Uncharacterized protein (Magnaporthe oryzae (strain 70-15 / ATCC MYA-4617 / FGSC 8958)) | 1.55487 | 0.0228293 |
| MG07_T12833-MG05_T10644 | Similar to OOW_P131scaffold01323g1 Uncharacterized protein (Magnaporthe oryzae (strain P131)) | 3.1251 | 0.00801844 |
| MG05 to MG12 |  |  |  |
| MG12_T03538-MG05_T09503 | Similar to OOU_Y34scaffold00050g19 Uncharacterized protein (Magnaporthe oryzae (strain Y34)) | 2.31816 | 0.0476693 |
| MG12_T10120-MG05_T05807 | Similar to OOU_Y34scaffold00694g3 Uncharacterized protein (Magnaporthe oryzae (strain Y34)) | 6.29854 | 0.0347445 |
| MG12_T10806-MG05_T10440 | Similar to MGG_13283 Uncharacterized protein (Magnaporthe oryzae (strain 70-15 / ATCC MYA-4617 / FGSC 8958)) | 2.55896 | 0.0328282 |
| MG12_T11764-MG05_T11041 | Similar to OOU_Y34scaffold00830g2 Protein kinase domain-containing protein (Magnaporthe oryzae (strain Y34)) | 1.82065 | 0.0140025 |
| MG05 to 70-15 |  |  |  |
| MG05_T11871-MGG_10001T0 | Similar to MGG_10001 Uncharacterized protein (Magnaporthe oryzae (strain 70-15 / ATCC MYA-4617 / FGSC 8958)) | 1.68659 | 0.0112608 |
| MG07 to MG08 |  |  |  |
| MG08_T06349-MG07_T08696 | Similar to MGG_16951 Uncharacterized protein (Magnaporthe oryzae (strain 70-15 / ATCC MYA-4617 / FGSC 8958)) | 2.9896 | 0.0361424 |
| MG08_T11585-MG07_T07835 | Similar to OOU_Y34scaffold00519g25 Uncharacterized protein (Magnaporthe oryzae (strain Y34)) | 1.61001 | 0.01958 |
| MG08_T12104-MG07_T12083 | Similar to MGG_04195 Uncharacterized protein (Magnaporthe oryzae (strain 70-15 / ATCC MYA-4617 / FGSC 8958)) | 1.51142 | 0.0363296 |
| MG08_T12161-MG07_T12833 | Similar to OOW_P131scaffold01323g1 Uncharacterized protein (Magnaporthe oryzae (strain P131)) | 3.1251 | 0.00801844 |
| MG07 to MG10 |  |  |  |
| MG10_T10398-MG07_T11513 | Similar to MGG_15985 Uncharacterized protein (Magnaporthe oryzae (strain 70-15 / ATCC MYA-4617 / FGSC 8958)) | 8.51519 | 0.0310333 |
| MG10_T11803-MG07_T12380 | Similar to OOU_Y34scaffold01027g1 Uncharacterized protein (Magnaporthe oryzae (strain Y34)) | 9.15927 | 0.0497728 |
| MG10_T12226-MG07_T12083 | Similar to MGG_04195 Uncharacterized protein (Magnaporthe oryzae (strain 70-15 / ATCC MYA-4617 / FGSC 8958)) | 1.50526 | 0.0422303 |
| MG10_T12478-MG07_T07472 | Similar to OOU_Y34scaffold00220g1 Uncharacterized protein (Magnaporthe oryzae (strain Y34)) | 3.08804 | 0.021882 |
| MG10_T12790-MG07_T12833 | Similar to OOW_P131scaffold01323g1 Uncharacterized protein (Magnaporthe oryzae (strain P131)) | 3.1251 | 0.00801844 |
| MG07 to MG12 |  |  |  |
| MG12_T03644-MG07_T08124 | Similar to OOU_Y34scaffold00665g21 Uncharacterized protein (Magnaporthe oryzae (strain Y34)) | 11.2494 | 0.0137724 |
| MG12_T04358-MG07_T07472 | Similar to OOU_Y34scaffold00220g1 Uncharacterized protein (Magnaporthe oryzae (strain Y34)) | 2.03881 | 0.0348794 |
| MG12_T06903-MG07_T08875 | Similar to MGG_10556 Uncharacterized protein (Magnaporthe oryzae (strain 70-15 / ATCC MYA-4617 / FGSC 8958)) | 9.18501 | 0.0365299 |
| MG12_T10774-MG07_T11161 | Similar to OOU_Y34scaffold00517g3 Uncharacterized protein (Magnaporthe oryzae (strain Y34)) | 6.23405 | 0.00652638 |
| MG07 to 70-15 |  |  |  |
| MG07_T07472-MGG_14344T0 | Similar to OOU_Y34scaffold00220g1 Uncharacterized protein (Magnaporthe oryzae (strain Y34)) | 3.08804 | 0.021882 |
| MG07_T11513-MGG_15985T0 | Similar to MGG_15985 Uncharacterized protein (Magnaporthe oryzae (strain 70-15 / ATCC MYA-4617 / FGSC 8958)) | 8.51519 | 0.0310333 |
| MG07_T12380-MGG_15031T0 | Similar to OOU_Y34scaffold01027g1 Uncharacterized protein (Magnaporthe oryzae (strain Y34)) | 9.15927 | 0.0497728 |
| MG08 to MG12 |  |  |  |
| MG12_T03538-MG08_T09919 | Similar to OOU_Y34scaffold00050g19 Uncharacterized protein (Magnaporthe oryzae (strain Y34)) | 2.31816 | 0.0476693 |
| MG12_T10120-MG08_T06466 | Similar to OOU_Y34scaffold00694g3 Uncharacterized protein (Magnaporthe oryzae (strain Y34)) | 6.29854 | 0.0347445 |
| MG12_T10806-MG08_T10717 | Similar to MGG_13283 Uncharacterized protein (Magnaporthe oryzae (strain 70-15 / ATCC MYA-4617 / FGSC 8958)) | 2.55896 | 0.0328282 |
| MG12_T11764-MG08_T11289 | Similar to OOU_Y34scaffold00830g2 Protein kinase domain-containing protein (Magnaporthe oryzae (strain Y34)) | 1.81954 | 0.0139821 |
| MG08 to 70-15 |  |  |  |
| MG08_T11585-MGG_10001T0 | Similar to OOU_Y34scaffold00519g25 Uncharacterized protein (Magnaporthe oryzae (strain Y34)) | 1.56824 | 0.0264035 |
| MG10 to MG12 |  |  |  |
| MG12_T03538-MG10_T10527 | Similar to OOU_Y34scaffold00050g19 Uncharacterized protein (Magnaporthe oryzae (strain Y34)) | 2.31816 | 0.0476693 |
| MG12_T10582-MG10_T12454 | Similar to OOU_Y34scaffold00254g3 Uncharacterized protein (Magnaporthe oryzae (strain Y34)) | 4.10664 | 0.0487841 |
| MG12_T10806-MG10_T09606 | Similar to MGG_13283 Uncharacterized protein (Magnaporthe oryzae (strain 70-15 / ATCC MYA-4617 / FGSC 8958)) | 2.64201 | 0.0367329 |
| MG12_T11764-MG10_T12105 | Similar to OOU_Y34scaffold00830g2 Protein kinase domain-containing protein (Magnaporthe oryzae (strain Y34)) | 1.81954 | 0.0139821 |
| MG12 to 70-15 |  |  |  |
| MG12_T03538-MGG_16930T0 | Similar to OOU_Y34scaffold00050g19 Uncharacterized protein (Magnaporthe oryzae (strain Y34)) | 2.31816 | 0.0476693 |
| MG12_T10582-MGG_17266T0 | Similar to OOU_Y34scaffold00254g3 Uncharacterized protein (Magnaporthe oryzae (strain Y34)) | 4.10664 | 0.0487841 |
| MG12_T10806-MGG_13283T0 | Similar to MGG_13283 Uncharacterized protein (Magnaporthe oryzae (strain 70-15 / ATCC MYA-4617 / FGSC 8958)) | 2.64201 | 0.0367329 |
| MG12_T12289-MGG_10001T0 | Similar to OOU_Y34scaffold00519g25 Uncharacterized protein (Magnaporthe oryzae (strain Y34)) | 1.46359 | 0.0421933 |
