# Supplementary material for: Genome-Wide Comparison of Magnaporthe Species Reveals a Host-Specific Pattern of Secretory Proteins and Transposable Elements
Source: PLoS One. 2016 Sep 22;11(9):e0162458. doi: 10.1371/journal.pone.0162458 (PMC5033516; doi:10.1371/journal.pone.0162458)
Supplement: S6 Table — (DOCX) [file pone.0162458.s007.docx]

**S6 Table:** Pfam domains of core set of candidate effectors gene families.

| **Pfam domain ID** | **Pfam Domain Name** |
| --- | --- |
| PF16541.1 | AltA1 |
| PF00188.22 | CAP |
| PF07249.8 | Cerato-platanin |
| PF05730.7 | CFEM |
| PF00187.15 | Chitin_bind_1 |
| PF01083.18 | Cutinase |
| PF08881.6 | CVNH |
| PF07510.7 | DUF1524 |
| PF08520.6 | DUF1748 |
| PF11578.4 | DUF3237 |
| PF14273.2 | DUF4360 |
| PF01375.13 | Enterotoxin_a |
| PF00254.24 | FKBP_C |
| PF12296.4 | HsbA |
| PF01185.14 | Hydrophobin |
| PF06766.7 | Hydrophobin_2 |
| PF01476.16 | LysM |
| PF02102.11 | Peptidase_M35 |
| PF01042.17 | Ribonuc_L-PSP |
| PF12680.3 | SnoaL_2 |
| PF13577.2 | SnoaL_4 |
| PF04099.8 | Sybindin |
