# Supplementary material for: Genome-Wide Comparison of Magnaporthe Species Reveals a Host-Specific Pattern of Secretory Proteins and Transposable Elements
Source: PLoS One. 2016 Sep 22;11(9):e0162458. doi: 10.1371/journal.pone.0162458 (PMC5033516; doi:10.1371/journal.pone.0162458)
Supplement: S1 Text — (DOCX) [file pone.0162458.s008.docx]

**S1 Text:** Unique protein-coding gene sequences present in MG07 compared to other *Magnaporthe* isolates.

**a. Venn diagram depicting shared genes in unique genomic regions of MG07 isolates with rice, fingermillet and foxtail millet isolates**

**
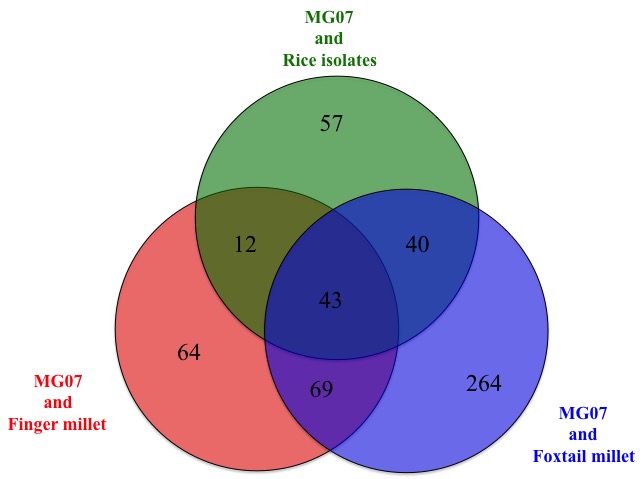
**

**b. Functional Annotations of genes uniquely present in MG07 grass isolate**

| **Gene ID** | **Annotation** |
| --- | --- |
| MG07_unique_gene_7 | Similar to MGG_08651 Ankyrin repeatl protein (Magnaporthe oryzae (strain 70-15 / ATCC MYA-4617 / FGSC 8958)) |
| MG07_unique_gene_8 | Similar to OOU_Y34scaffold01147g1 Arylesterase/monooxygenase (Magnaporthe oryzae (strain Y34)) |
| MG07_unique_gene_11 | Similar to OOW_P131scaffold01677g4 Uncharacterized protein (Magnaporthe oryzae (strain P131)) |
| MG07_unique_gene_13 | Similar to Uncharacterized protein (Magnaporthiopsis poae (strain ATCC 64411 / 73-15)) |
| MG07_unique_gene_15 | Similar to MGG_02273 Uncharacterized protein (Magnaporthe oryzae (strain 70-15 / ATCC MYA-4617 / FGSC 8958)) |
| MG07_unique_gene_23 | Similar to OOU_Y34scaffold01040g2 Uncharacterized protein (Magnaporthe oryzae (strain Y34)) |
| MG07_unique_gene_28 | Similar to Uncharacterized protein (Magnaporthiopsis poae (strain ATCC 64411 / 73-15)) |
| MG07_unique_gene_35 | Similar to OOU_Y34scaffold01193g4 Uncharacterized protein (Magnaporthe oryzae (strain Y34)) |
| MG07_unique_gene_37 | Similar to OOU_Y34scaffold00305g1 Uncharacterized protein (Magnaporthe oryzae (strain Y34)) |
| MG07_unique_gene_41 | Similar to MGG_12203 Uncharacterized protein (Magnaporthe oryzae (strain 70-15 / ATCC MYA-4617 / FGSC 8958)) |
| MG07_unique_gene_42 | Similar to OOW_P131scaffold00088g4 Uncharacterized protein (Magnaporthe oryzae (strain P131)) |
| MG07_unique_gene_47 | Similar to MGG_00241 Polyketide synthase (Magnaporthe oryzae (strain 70-15 / ATCC MYA-4617 / FGSC 8958)) |
| MG07_unique_gene_48 | Similar to MGG_06544 Uncharacterized protein (Magnaporthe oryzae (strain 70-15 / ATCC MYA-4617 / FGSC 8958)) |
| MG07_unique_gene_54 | Similar to OOU_Y34scaffold00305g4 Uncharacterized protein (Magnaporthe oryzae (strain Y34)) |
| MG07_unique_gene_59 | Similar to MGG_16989 Uncharacterized protein (Magnaporthe oryzae (strain 70-15 / ATCC MYA-4617 / FGSC 8958)) |
| MG07_unique_gene_62 | Similar to OOW_P131scaffold00513g1 Uncharacterized protein (Fragment) (Magnaporthe oryzae (strain P131)) |
| MG07_unique_gene_67 | Similar to OOW_P131scaffold01475g1 Uncharacterized protein (Magnaporthe oryzae (strain P131)) |
| MG07_unique_gene_68 | Similar to Uncharacterized protein (Magnaporthiopsis poae (strain ATCC 64411 / 73-15)) |
| MG07_unique_gene_70 | Similar to OOU_Y34scaffold00857g6 Uncharacterized protein (Magnaporthe oryzae (strain Y34)) |
| MG07_unique_gene_72 | Similar to OOW_P131scaffold01206g1 Uncharacterized protein (Magnaporthe oryzae (strain P131)) |
| MG07_unique_gene_73 | Similar to OOW_P131scaffold01369g1 Uncharacterized protein (Magnaporthe oryzae (strain P131)) |
| MG07_unique_gene_75 | Protein of unknown function |
| MG07_unique_gene_76 | Similar to MGG_02583 Mitochondrial ATP-binding cassette sub-family B member 6 (Magnaporthe oryzae (strain 70-15 / ATCC MYA-4617 / FGSC 8958)) |
| MG07_unique_gene_80 | Similar to OOW_P131scaffold01579g12 Uncharacterized protein (Magnaporthe oryzae (strain P131)) |
| MG07_unique_gene_87 | Similar to OOU_Y34scaffold01115g7 Uncharacterized protein (Magnaporthe oryzae (strain Y34)) |
| MG07_unique_gene_88 | Similar to OOU_Y34scaffold00863g4 Uncharacterized protein (Magnaporthe oryzae (strain Y34)) |
| MG07_unique_gene_100 | Similar to OOU_Y34scaffold00517g6 UDP-glucuronosyl/UDP-glucosyltransferase (Magnaporthe oryzae (strain Y34)) |
| MG07_unique_gene_101 | Similar to MGG_14722 Ent-kaurene synthase (Magnaporthe oryzae (strain 70-15 / ATCC MYA-4617 / FGSC 8958)) |
| MG07_unique_gene_108 | Similar to OOU_Y34scaffold00335g2 Uncharacterized protein (Magnaporthe oryzae (strain Y34)) |
| MG07_unique_gene_109 | Similar to OOU_Y34scaffold00869g2 Uncharacterized protein (Magnaporthe oryzae (strain Y34)) |
| MG07_unique_gene_117 | Similar to OOU_Y34scaffold00519g13 Citrinin polyketide synthase (Magnaporthe oryzae (strain Y34)) |
| MG07_unique_gene_118 | Similar to OOU_Y34scaffold00378g1 Uncharacterized protein (Magnaporthe oryzae (strain Y34)) |
| MG07_unique_gene_124 | Similar to MGG_14377 Uncharacterized protein (Magnaporthe oryzae (strain 70-15 / ATCC MYA-4617 / FGSC 8958)) |
| MG07_unique_gene_126 | Similar to MGG_16025 Uncharacterized protein (Magnaporthe oryzae (strain 70-15 / ATCC MYA-4617 / FGSC 8958)) |
| MG07_unique_gene_127 | Similar to MGG_06325 Vacuolar membrane protein pep3 (Magnaporthe oryzae (strain 70-15 / ATCC MYA-4617 / FGSC 8958)) |
| MG07_unique_gene_129 | Similar to MGG_03036 Uncharacterized protein (Magnaporthe oryzae (strain 70-15 / ATCC MYA-4617 / FGSC 8958)) |
| MG07_unique_gene_130 | Similar to MGG_07833 Ferric reductase (Magnaporthe oryzae (strain 70-15 / ATCC MYA-4617 / FGSC 8958)) |
| MG07_unique_gene_131 | Similar to OOW_P131scaffold01764g3 Uncharacterized protein (Magnaporthe oryzae (strain P131)) |
| MG07_unique_gene_133 | Similar to Uncharacterized protein (Magnaporthiopsis poae (strain ATCC 64411 / 73-15)) |
| MG07_unique_gene_147 | Similar to OOW_P131scaffold01477g1 Uncharacterized protein (Magnaporthe oryzae (strain P131)) |
| MG07_unique_gene_150 | Similar to OOU_Y34scaffold00260g2 Uncharacterized protein (Magnaporthe oryzae (strain Y34)) |
| MG07_unique_gene_151 | Similar to COQ4 Ubiquinone biosynthesis protein COQ4, mitochondrial (Magnaporthe oryzae (strain Y34)) |
| MG07_unique_gene_152 | Similar to OOU_Y34scaffold00316g2 Uncharacterized protein (Magnaporthe oryzae (strain Y34)) |

**c. Protein sequences of unique genes present in MG07 grass isolate**

>MG07_uniq_gene_7

MTPHQDYTVGIICAIGFEMSAVRYMLDKEHQRLHGKDGDPNIYTLGELSGHNVILACLPG

TQGKGAAATVATNMARSFPSINWRFLVGIGGGVPSSKHDIRLGDVVVSMPEGPYGGVAQY

DLGKDEDDGFRLKGFLSAPPPRLRNAVEMMRSDHFVQENKIEEYLSIMLKRGSRLKSLYS

RPEAHTDILFDEADSHASICSTGGSCDKTKVVPRLPRDFEGPGIHYGLIASGDSVIRSAA

KRSQKVRDVGDILCFEMEAAGLMTEYSGLVIRGISDYADSHKNDGWQHYAAAAAAACAKE

LLTYVDPAASPRTSSSDTDGSLRNAAAAQNKPGA

>MG07_uniq_gene_8

IYFIFEVYIGINAPPDRAIIFYFGGGFIIGNADNNINFTANIIIQIYNYIFILNYRFAPE

YPAPAAVKDVYATLKWVQTYTAKFGINTERVVFFNINVGGEIATGTALIIYNKNLTNSKK

FPLPAGLVLRYFILNNRTVGSIENPKYYYYIWNCVINKIIWIIYAGGKKKTEKTNDIIFI

YAALTRIGPDKLRGFPAIFMDVGELDLFREKITKFITALATVGVNIEFYYYSGFLYGVKI

ITPEISKIIIINKNTCKFIVIDISNNGEFNEAENVYF

>MG07_uniq_gene_11

MSGEQFLFSAELICPEVQATLPKRYSIRPLRRSDYSSGYLETLSVLTEVGEISRKQFEER

FDEMVRRKDTYYIVVIEDTTSKKVVATGTVVVELKFINGISKAAHLEDVAVAKECQKMGF

GLQIHRARDFIVRQTSCYKGVLDCREYNVGFHEKAGYKRCGVYMKKRYDGVPVEAFLPAS

S

>MG07_uniq_gene_13

MTDFGPETTAAAVVEQFTDQVQGRTFVLNGVSPGGIGAEIAACLAQANPSSLIVVANDNG

STTLQSLLGEVNGPNASVAISVIAAHPSSISGVRQAAQDIIARQDVENIDVLMNIPTEMP

RAYTETEDGLEYLLQTNYLSQFLFTNMIMPKLLKAQRPRVINVSSSANKIAGMRWHGVNF

KEAGSYEPWTAYGQTKTAAILFTTALNAKFGGQGRDLRSYAVHPGGVRTKLQEQLSSHAL

ERAFEGTKQRFGEETAKEFFRWKTLSAAGSTPLRAALDPSLPSRAGIWLEDCKLLEESIH

LHVRATNLVDANRLWDLSNLLLSTHF

>MG07_uniq_gene_15

MLFTQITAIFGFAANFASAVNIKLYSGSRTCSGFFIVCSNIISDRCYTSATNFRIIRFNG

GNGIRNFRGFINGCDNIIISQNTGIFFYIFRGDGGNINGAQWRAASLFRRGPEKENCPAD

QGGFKKVAKPNRVVFADRAKYDVKDLDNIKLTKLVNN

>MG07_uniq_gene_23

LSPKEKEQKCERARDRLLNAQSNLHLVSAVYQIPFGTIPQSSQAWHDFNNAVSSSEPGRS

LRCVLAVSEKWGVDVAKHYAFLSKGRRYCDVLRTAANKVPNWKLAARLLSYRIFKRINVY

RVECG

>MG07_uniq_gene_28

MNAQNSCGNPDLISNCPSNATVLNAFLNSPVTWEKIEYLARRTSDVIACGNNSGQNANQF

DQGCIPKLDQFIASLVLHSKVQSSTLMCSLVYLGRLKVRLRDCKGHRSTPHRIFLASLII

TAKYLNDKSPLNRQWARYAFLGTIDAGDGNLGPDFHFCTAGVNVMERQLLDLLSWNVEIR

KSELYNELELFSRPTCHYISKRITNAKWIPASNPTAKAGRIVIEQLPLNRDRGIEQGFV

>MG07_uniq_gene_35

MVGQGAYLYRIEQMHQKYGPIVRVNPQELSIKDPSYYDQLYVAGAVRRTNCWTGNGGMEF

KDSHGTTVEHDHHRLRRKPMEPYFSRAGISRIEPVLKRIVQTLVDRLDQYRGSGNVISLD

CLFSAFSGDVITAICVGDGDATSLRHPSFDPEWILMSLPERFLEKANPEFKRLNEWRNRA

GKHIMEAMMVQESNDASDLLHKASSRHATLFHQLLSPQSGLPDSERSIERLTTEAQVLLG

AGTVTTTRTLSYLITHILLKDCVKITLERELRAQAVNLRNGSTSLADLEKLQYLQACVKE

GLR

>MG07_uniq_gene_37

MEKYNIFIRCDPEEIRYILFKNGFNNVKKRAIIFPINNWLLENDGKKIGKWFNFVFTNNL

LPIFFLFLVRIGNNKIKKINNLINKTSEKLRSQIYTEGVYYML

>MG07_uniq_gene_41

MAGNHSTAFPKVDGITVFVLPPEGYKVDFEHPAKQSAMEHFLVFGIVGYLALMALCQRLY

TKIFLLCSVVMQSSQIHSIVSGGLCHHTWEMPIDVFEYSLI

>MG07_uniq_gene_42

HSPASSARHRVSLANLAKTKTLFILTNGGFFVCSATHKFFDVFPSGQPDKAFPPTTYDAI

SRHVLPVPNIHKSMYETLKGDDGKSFVTSGPNMKMPLPEETRYTYIKPYNQFSTEKVVFA

YTCERSNGITINNMRIRIDAFLQSYLHKDIFGSTLALNLDGGQSIFIGFQKLGQSMKVLT

QGGLNAEKILGIKPVNPGTVREVSTMVKHELISSARATGGHCVRRPDSLQTATAGRRDAT

GGSAAVVYTNGNCASRMALRSIYEMAKCTIDPPQQNRDRSKLPRADIVGGLPAEPLTLNP

APFLLFWWQRLRHSQDTLTGEQSLWQPKLDRDRVKTNVLCASTAIITATLAVVTAAIGEI

>MG07_uniq_gene_47

MISTTTGCRAFHDDADGYARGEGIGVIVLKRLEDALAGNDSILGVIRASARTYSTTTTSM

THPSHESQERLYRRVMAKAGLDPTEIAYVEMHGTGTQSGDLEEITSVINESAPPGSRTRA

NPLTVGTLKPAVGHGEAAAGVMAIIKVLLMMRDRVIPAQPGWPFKINQRFPPLDPLNVRI

ATQHYPLKPSPKGDKRVKIMLNSFDASGGESCLVFQEPPPSVGGRTQGSSDPRTAHVVTL

SGRTTTSLAGNRQRLIDWLEANPNSRLEDLAYTTTARRMHEPLRAAYVAGSVDELVRKLH

QASAKGCEDPKAKPKPAGRIFLFTGQGSEYSAMGSVLFKTNRVFREKMLIYERLVTHMGM

PPFTEVITNPEFLQRATPAQTQMATVALEIALTETLAQYGVEPTMVVGHSLGEYAALCAA

GVLSVADTLFLVGQRALLMEKHLLQHRLQKNAPELSIASGTINQIQTLEAHMKSQGCRTT

VLRTKYGFHSSQVDPILDELTGIAAGVEFKAPVVPIVSSYTGQIVPVGDQTKFGPQYLAR

QCRGTVNFVGAIQAAAAAAAGEAASMHKTLWVETGPDPVLLGLLRKTLPEIDSKNLLPCI

KSKLDDNWTSLPSVLKSAYESGVEIEWPEFHRDFKDHVRLLELPLYAFDFRDFWHEPFKG

PKETTIQSAEAGKAHNCSCSQPPQTSAASRFPASFPGFPTTTLPTVESEDVDAANKTISV

VFAADTSEPELLRAIEGHVVLNHVICPMSILVDMALTAAKYCHFRLHGEAKIPAMSVSNI

RMNHALVLHSNVARKPIIRVKTVMRKDQSSADVHFSWLKFSSDEKADPVEEEGGSCTANF

ETQEEWPASVNSSLFLVKSRIAALRAARLEGRAHGLLKPVIYGMFAQTVNYSEPFRGIDE

VILDLNCQDAIAKVTLLPDASSSGSFVMNPFWTDALTHLGGFFLNSGLRYPQQDLLCMAR

GFDLWRVVGLSGTAMQPGQAYTNYTFMQDVEGNFVTGDRYIFDSNDNLVQTLLGLKFQKL

KRSVMAAVFGTVPSTNLKAAGRSCSTNQHDDPRLSIKVNNHEADAISCAEATRRSAPSTP

CSSPLSPLSGMSADCHKPDAANLMKTIMAIVSQEAGCRLDELTDETAFADLGVDSLMGIS

ILAVIKRETGVDLDSTFFIQFETVKDAKAALELQSAQEPPTVSHTSLQQISFKAYWPPTP

PPEQKESKVEAPNCSPIPPMNATGDAVMSDSSTPPLDGAERESTSTQGPTGTASLVHFSG

PRATPVQNLFLLADETGSSLQCIQLPAATTSNGSPVSAWGVEPKFANKQIDPESHSVHDL

AVVSLNAIREKQQSGSAPFLLGGIGAGAAVAIEVARLVQLESLRDCLGLVLLDPSLTSDA

VERASSARDVRLHPAKRDLCSAMAQAVGSYSKDVVSQPPLEIEAVAIMSESSSAEQELLS

QILPRMRVKTSETPTEKGLLMRAPALANTNRSFSEAVRELC

>MG07_uniq_gene_48

MALAGSPTADFLHPSLPSPVLGLSDASSIVLLLLVFGVTYQTCSIIYNIFLHPLRNVPGP

FWSRATRVPWALQSVRGTQPFRTKEAHDRYGSVVRIGPDHLSFTDHRAFQDIYGHRIGVK

KAVPEMRKAGIFFDPPFPEFSPSIANADRENHARLRRALAHSFSEKELRKQEELMNFYLR

KMVGRLCALSAEGPVDIESWVNYAAFDITGEMAMNLHFNCLEQAEFHVAIKTILDMLPSS

VVSLAIQYLGFEKPLKAVFRMAGGVQAAKDLRKELDKMLIYRLQQTDMKDVMEELISRND

FLGLDYEGMVAHALAFLIGGADTIIAPLTGAIFFLLKNPDKMEKLKQEINGRIKSESDIT

IEAVMSMPYLIATLKEALRLSPPLPAGLVRVIPGGGNEVAGVWSPAGTMVEVQQWAMGHC

ASNWENAQQFIPERFLGEDLDDNLEAYQPFSVGPRACLGRNLAYIMMRLTLCHIVYNFDM

KLAEDSQDWFQRRRNFFAWERLHMNIHLSPAR

>MG07_uniq_gene_54

MSQRLPKFWRIAKVMSKGSGIWKSLRLTCAKIANPVRGLTLKLPGARSGLGTGLHVL

>MG07_uniq_gene_59

MVQTYFVINLLVAFTAATIPDIVYRGVGSNPDIVKGWGGFKCRGGDFGEPKDHSMDLYTH

VTMNPDSPGHNTDPWILTSKSYDWVKGYMGRHKKGRTVHIYHIRTKGLKNIFDIADYCKA

KGLPYPRSVEQEVSVKDFIPWSNIADWDSYHVSNDGQVTPMPKYTLGTQKN

>MG07_uniq_gene_62

MSITKTAMVYLVNHVFLPPRFPNGDDWESIHDSTMQSTLLASIKRFEAHIAPGRLTLVRS

AAAMIRRMIESQDENGRVHLNKLEHTLHE

>MG07_uniq_gene_67

MSIPVNASAIAQADQHTERLQQNLLVAMVDAGVPVENCRPSQREAYQSFIGQQAQINSDN

HVQMRSPSLQAVRAYISAERAFGRGPVEDFAIPAEMSSALGISASAPTPSPRLSAAISAR

GGPRPVGFRGLSGEAIVMLRKALADAAVYRDKNKKYLSPQIQIGAQFRTAGVDRSNKTLE

CLANASTSTLDEPIIRPSLVNPHQQSGTTSIGQVQAHRNNNGGERGLTRP

>MG07_uniq_gene_68

MVSSASLRLRGIATAKPVAADDSKDFELLILKHPHHNVETSFWHGSVVRVLRKRSVPAKE

ELQTGVPIEKQGGLCSLIEKFGVAEISLVVANAALLYCGHQESQLPPYRLRGSSMSLKAL

LEGLRPQVMSGQQKLALQLEIAKAFWQLYASSWMRRTWTNEVVHFVFQRQKETPSHQEIR

VDDPLLQVQFDSATMEQLEIGQQPYFPWPAVHSLGVILLEIELGDCINRTVEDLGYLDPE

TGWPKPHAEFKAAEILIEYEKLLEESVNPGACSNVAHIIKSCLRITSDLNQLHDRGIIEQ

RHAIFKLIVSPLETLLNKKFRGREVIGLLIGEWVSNLRTVQQITNTSRSDLSSHKWFKRL

DQLTDILTITPEDRDGNSRATRVALLDTGISNEFQHKPDLREPIRGYYDFIAGCNSDYQD

ATGHGTKALKLLLKVYPDAHVYIGRIWGDGFNDEVPGRMAQAIDHCRKEWQVDVIVIPSG

FAKNYRCVSKALTEAGAANIVVVAAAGNHGNLDDVVFPGRLYTANKLLCMFATNALISLS

PMWNPRVAAGQGIKGCYAILGENICLQTDTDSDNCQGCKGLLLSGTSYSTVIGAGVAAHL

LDFSGQPECRNSIEERDSLTSVEGMASVFSSMCHSSNDEVPCMRPWMLLPKGVQGEFGED

MWTFRQRARKRICETISTALEKRY

>MG07_uniq_gene_70

MSSCVFYAAYLGFSDLIYHWIEKDGVDVNHAGSSRRTAILVAVSRGYLSLMEYLVSKRAD

VNISSNEGWTPLYAALYKGHLEVVKLLFEKGADCAVENNDGWAPLNVTSNNGPFEIVKLL

FEKGADWAAADNGRRAPLYNVLKNGHLDVVKFLFRHGADLTMANNHRRTPIFAASSNGHL

EVVKLSKKGRLQMFR

>MG07_uniq_gene_72

MNDRDLLSWLAAIPSHHLAPLSDKRKRKRKRAAAPPTPPPDSCSSSTSSTHTTSTPAKRP

AIHVYDDIDQTPRPARSRPAEESLTSTPSKRSRTSQTSFASGSKRIREAESSGDLCSQSF

SRDDTDALPEALRTLQTDLEGACVGAPIISSSRKHEISAAGALGKSIRDFHFLPPDSGQV

ESPTFRDVCKYQKRAAMYNDDRFEEAAWSACIIIPLLELAISDIKELAIIPCTTAKVADK

DLLPRGYSNCKMVDICLALEPSHVPKTLSAVRRVQHLAPSKTINHTDYHPLSYRPIGLSI

EVKSPSGSTVEAVEQLAVWQYAQWKMLEILAMNPDDNQPLALEGFDFLPGISIVGPKWSF

SATIRHRDGSVVQWKGGKLGDTTTAHGIYSIVWGLRRIAKYLTETYWPWYEKEILRMARI

KV

>MG07_uniq_gene_73

MPIPSIPKFLIQMSGAPGSGKSTIAVLLRPHFNATIIDHDVLRSNFISIPVFQFDQAVKQ

AYALQWELARDFIKQGVDFIIIDSTCNYPEVVAKGFSLAVKYEYRYWYVECNVRDINLLD

KRLRARTPKANQRPAVDCPPESAQGNGVQVGENARERFA

>MG07_uniq_gene_75

MPRHFLHWKKTLKPLTIRGISSFQPRPNHCQSLPRLVGNGLSNCHYKHLCFVGLWLGN

>MG07_uniq_gene_76

METRDFTGSILEFVDAVLDIAPLQRILEKSSVSARQTKSKSLNCCNGNLRLVNVSFTYPG

LDKPVINGLDVIFRPATKVAVMGPSGAGKTTLLKLLMRHLSPSNGFILVDGQNIQNMDKT

SFHKHVGIMPQIPYIFNTTIMENIMVGNASATIEEVYEACRNATIHDSIMARPDGYKTEI

GEQGGSLSGGEKQRIELARLFLKRPKIALLDEPTANLDEKTETQVLCNLFSQFSNATIIM

VT

>MG07_uniq_gene_80

DQSLCGNSNEQAAGACLDSENKPDIDPDAVPLESILNNAQNTAPTSFSGVSPNVPPSSLY

LAVTGVSDEALWLQPAASAPPTDGLGDALPSFGSWYPMPCEPFLQSFYENAPIPAHFSTM

ATTTSDSGFAILPYSNAPAGFDCFAPRCAPFETVACFDMLVPQYQHQWVLALSCTKRTRQ

EAEELGFKKVLLSSDLPRITASVHDADGLSKPHNQQSEHITAILIFSTPHDWCLDLQLAL

DLLLPCNHAASGSANGSFIIDNGAYGNPPKLYFRSLSSGEAESEFPSRLGVEDFKCAFQS

IWLKKVGLLGFEKSDGKI

>MG07_uniq_gene_87

RNPYKNLLPVWISPFNTLDLHQPPLFASNLNRLKNGCKFKILEIVREFQKTNKNLQSEII

IIFRFKDRIGGVKAKIAIVEAKTKLWEKTISRLKKHYIRIKNSNLLRAIRSFWKKFTNLY

VEYNALRRQINNLETRLHILKKICGNNNRSAKKIMHKLHPKKIKVSIYLPISPTDKLGHL

LEI

>MG07_uniq_gene_88

IAIVENKCIKVDNTHNSLNNNLISPQLNTDQWQILIALHRTLLYEHHDFFFVSQHPLTNP

ALRRFAIKYAIPARIWRYGIYNLMVIENDDLHVKKVWKAVFRQWYSRISDMAPTTGRLYH

HL

>MG07_uniq_gene_100

MTLATTSNATHNGHLAETNEATSVPRPYLLICTTAASGHFYPTLQITEHLIKQGFEATVI

TAEHFRRQVERAGARHVALPPFIPSAQALEERKQLTTGFQNMVWGLTHVVVKRIPDYHAV

VVSTLESLRAERPGQQVVVLTDLIFGGVNPLLHGAPPPRGFAVRPPVLAYNVVPLALPGV

DVGPWGLGLPPDSTEFGRARNRLLHEAYLSPGGVFGSYCREYGRVMEALGAAPPATDAIT

AMTTCPDVTLQACSPSLELPRSDLPAKIKFLGCLPCKTPDPTFEYPEWWLRDVISSGRPN

RRKIIGVAQGTVAVNYNDLIIPTIQGFRGRDDVFVVAVLGVRGACLPAQVEIPANAKVVD

FMPYEALLPHLDVWVINAGYGGFTQGVMYGIPMVFSGDTEDKAEVSMRGQCAGVGFNLRT

ASPTPEQVAQGVDSVLQDDNYKARVMEIRKENEAMKSLEGVEKHVWDLARRSQAL

>MG07_uniq_gene_101

MHSIALPHPRIVDQQASLLAQHLLHNYDPDQGAGSITYSEQSQVIWTLLQGGFSGQQLGS

SVTEVARIWDQAPFVVTDAEDVAKTLVVLNKIGIPKSASGTMQEFQTGSNEGSVSTHCNI

LLALLHQPSPTEHLVQISEILGHLCTSCWKGAWPIRDERNSPPLYSGMLIVQAMGSVLEL

VENGRLPESILSNTETRTEIALALYHTVLLTIETQKDEGFWDGSMEETAYAVILLSVASR

ITLFDAVRQQLINAVRKAEKWLVPRSSPAGEGIWVPLGKGICGSPVLNRANRLAALKAAS

LLPAASTIGSCLQTANAANEMGPYIKLFRATPMFSAFPQERLLGAAVEASLFLPLLKEHR

SDVFARENVAKDRYFPLIPFTWTATCALEGLRPSPEFLWELTKMGLFLFQCDEFLEGLVQ

HEFADDLGVVARYVRHLIPLDPGDSGTTDEATGLFPGAERLRPLELMVDFILRHRAVVAA

TPGDRLSLARELRSYLLTQIQQTEINVRVAREGAAFAPAMSFFGWLETVAADHAASPLCF

AFIACLVPSTLCPELAGADVLPTAEEKYYSAAVSRHSGCMCRMQNDLASVARDAAEGNYN

SVDFPEFNATVAETKKEALRELAEYEKSAWLNALEKLEVVSRRRVAGLGESVKEATERRI

KCWRMYCNIVELYGQLWIVKDMSSKVTQAAR

>MG07_uniq_gene_108

MRKLEITPAIPSTLKIRINKIAETEIFQNSFTIYFTQPLKPIQIKQDIPGTNNYILAIVT

AKPYALIALKNTVVPLISIVEPKIQNEGLKAITHYKFTRMDSIINAIIRGLFVNILGTEK

LIRYPFQKPSQTPNTEYNY

>MG07_uniq_gene_109

MSASTGTDSGLSAEVVVGIVAIVVALPTAILVVFQIIQRTRRARERAPIQSATADVPPPA

TLRIDHSYRRFIYATFEETWVVGDMHAHPSRERDFIPLNRFFDASQGSDTPPPPPAPHVA

ADTWFDELDARYWGNKPYGMK

>MG07_uniq_gene_117

MSSTSPKATSAPSTAFFCPVPRLVDAAYLTEIRRQFTAKPFLKPLIGAIKETPAIWDLLA

RDKRAQHVASGARGDKFAKGLTKWMTEGETGLIASQPSTVVSFPLLFLTGLSQWCSYLDL

SGQTHSEAIKGLAAGGIQGYCGGMLSTVAVASASNEEELGRNAENMYRVAFLLGVFMSEG

EDGPPEHAPDMMIVRLKYPGQDLALLEQFPGVYASATTNPLTICFAGKKSELMRLKDWDG

EQLLGQTPRELARNVLGNILCGRCEWYKVVEAVAQRIRGTPSHSFLLLGGYDCVPVEPFH

HASLKISKIDLAQTLGELAGKKAELASYGDDTVVIVGSACRLPGATNLDELWGVLESGVS

KCEEVRPDRVPISHSYRANLDTKFTSGRKWFANLVDGADQFDNELFGISRSEATWMDPQQ

RVLLELAFEALDSYGHLGHRSYSREAGENVGCFIGGTLLEYNEHTSTHAPTSYSAIGTMQ

AFQCGRISHHFGWYGPSETIDTACSSSLVAISRAVSAVRSGECSMAVAGSANILGGLNYY

LDLGKARFLSPTGQCKAFDAAADGYCRADGAGVVVLKRLKDALASGDEILAVIPAAGTNQ

GGLSKSITLPDPRAQQQLYRRVVNSAGLQPKDISYIECHGTGTQAGDPNEINGLRSLFGD

GHREKKLYIGSVKGNIGHAEAAAGVTGLMKVLAMLRQSKIPPQASLENINPKIGNLEPFN

MAIPCGTVQPWDAPDNIRRAMVNSYGAAGSNASLVKKKNENKNDNAAALTTWPVVVSAAT

QASLEANCRALANMIRLEKPDLNRLLWTLSEKRQRHRFRVVLPATSNQDSDTLAVALEEA

SSSPAHSTPKKQQPVVLVFGGQSQRSIHLDRHLFDTTPRLRHHLAECDATLEELGYPRIT

GDILNHGKVDQDVLLLQTATFAVQYSCAKTWEDAGLRIDAVVGHSFGELTALAFSGAVSL

RDGIRIVAERALLMQNAWGHEKGKMLAVHTAVETVQKMVDAVPGLEIACYNSPAGQVVVG

SRKEIQAVEAVIAEKFKGTKCQHVDVTHGFHSKFVDDILPGLDKVDKSLEFAEPRIRLEL

CVDNSHLGKLSPGHIGRHARNPVFFTDAIRRIAQDLGPCVWLEAGTDSPIIPMTKRAVPD

PGAHTFLAVSFKSSRRPETVMSEVTMSLWRSGVDTTYWPWVGVTGIKPVRLPVYQFTRES

FWANFVGHPAELKGRLEEALQQQQQQQQ

>MG07_uniq_gene_118

YSVQNYNGLPELSHSRKNFENVNGDKIIHQKFLELFRRHGVDKTFGVAMVHRHFALERGE

HMVEFNGTSTPWPSIDGLDEGLVVTPTTWQMTKEGVRPYEFKLKAGTDAAQEIDFENPKV

QAFAKEFMEIVDSAGANGLFGLCDYPGDDFAGRLEVTEGRANINLPLKPFAHLRNVTDAA

WFFSPELYDEGGDGCRCRCLVNSDGSHKDSHQHDSSR

>MG07_uniq_gene_124

KKLVKNGEFKILFKIIKLISNKIWFFLEQFRNLKIYVIIRRKISVLKTDETYLDNGEKTN

F

>MG07_uniq_gene_126

MPNTSCLSTPSSKAQILLQNKIRKRRKTIFRKLEELRLCGARIYIVLEQNNRYFTYSSEK

TKYWPPSPRTLERSYPLPKEYAPGTAFND

>MG07_uniq_gene_127

KRFPQVHADRHLPSPPLDPSKKRLPKAIPLPAIMWKIPSGLYKVTRSLGPPASSTKIATN

NGSRARPVSQGLKRLTISKRQLMTDGPWRLLAPALRNYITFPSR

>MG07_uniq_gene_129

MVTDSKTVNRLPTQQSSALKTEIFCETYRKSHHKMHGVIAIAGATTGIGAAIVDALSEKG

VTDFVILSRKPGSDERTIAVDYNNVDNLQQVLEKNQVETVICTLSISDDSSGQAQMNLVT

ASDRASCTRRFMPSEFGMLYKEENLGHLPAYLWKLKAKDALAASSLEYTLVSIGFFLDYW

STPRIQTRIANAPPMFLDFGNRFASLPGDGSTKMVLTHSRDAGRFTVALLNVPRWETRYS

IIGNRLSLKEAVENAEEVLGTSFEVHYDSITDLEEGKVTLTPGLESMVKGTPMEGPLRFL

ASNTGLLQYKGQMDLGLQNNLVEMFPNIKPLTVRDVAKAWR

>MG07_uniq_gene_130

MPGAAGGKTGSRHSYVMRAKAGETKTLALLAAKKLTGAAETAVEKTTTRSSSSSPTAPTV

SVILTGPYGQSIMHSFRPDANVLCIAGGTGITFILPPFLEIIRNAPVPGRRVELAGTHDL

HIDVFVTRDVKGRAAGQGGKEAAAARNEVNAVSGFSKNSISSGEEVSEDANIAVHRPARV

EDPEVRRPKMDAVVDGFLGGVASGSTTVYASGPLEMVGDLRAAVARQNSSGKVWRGEERF

NVRLVSDNRIEW

>MG07_uniq_gene_131

MPAAHFEFLNRLLVPLALKFGITGYTQIRAFGEILPQDAGEDNPASNFPTSFDGIATFRY

PNDTALENMLAHPYYTKVVAVDEARFIDQEAHNGGQVAVFISATYDVVGNHAPSRNVWNG

DKAIKDYYEALFKKYDRL

>MG07_uniq_gene_133

MSTRTQHQLLTPPSNDDLDSGWLLASPVLKEDSSHFNAWSNTSFAADDDNGSHFAAWPDT

TFAAADGNDSSDLVVSPDTSSAATQNDENINFDDWLLFPRDGLTIKIMNTSSELFLTIKR

DVPLKKLMKASCKHFNLSLNLVRFLLDGHRTQPTDTIDTLEMSDGDTLEIHVEQIGGGNA

G

>MG07_uniq_gene_147

MVATVGSVSELSATTSCPSRSCVHKKPVVAENSFGWHGQESKVLVLAPGGDVGAVVDRQG

PDGLSLSIASPGSV

>MG07_uniq_gene_150

MRFSVPAAIFCALCSTVTATPGPGTTHANLQARQPISKDGKSSAKGKLNRAAPCAKEEEY

CRVAADCCGALLCGENGCFKLKSLNPRGLMD

>MG07_uniq_gene_151

MQVIRSLRSFPPNPFLSHSRRHFSVLDRPPPKYPGHVPLTRLERAALAVGSGLWSFIDPR

RGDLIAAFAEVTGTDHFLPRLRDAMLSHATGRRILRDRPRITSTSLNLPYLRSLSENTVG

RVYVSWLDREGVTPDSRAPVRFVDDEECAYVLQRYRECHDFYHALTGLPVVREGEVALKA

FEFANTLLPMAGFASLSAFTLKPGERERFLYTYLPWALSNGLRAKEIINVYWEEEMERDM

GDLRHELGIETPPDLRDVRRRERLSKQHPKD

>MG07_uniq_gene_152

MSANHVSVLPVTGRATSVAGTHTGLTAKAFALLLLLSFSIKPGGSLRIGLLKLAGSTKLR

WDDNGYPLEDPASSADLKSDIAQLLCSKAGLERAFDELYAAGVASLASDDPPTVTITATG

YEMLAGKCEEADIGSWKRQALIVACRAVSFKYLGFPNTVEERTSLKSYLEHTLKALRAHY

RGFEDLSPFTRVEVALCLIEASRFPGMAWKHMLITRRKDI
