# Supplementary material for: Genome-Wide Comparison of Magnaporthe Species Reveals a Host-Specific Pattern of Secretory Proteins and Transposable Elements
Source: PLoS One. 2016 Sep 22;11(9):e0162458. doi: 10.1371/journal.pone.0162458 (PMC5033516; doi:10.1371/journal.pone.0162458)
Supplement: S2 Text — (DOCX) [file pone.0162458.s009.docx]

**S2 Text:** Protein sequences of unique genes identified in neck infecting *Magnaporthe* in fingermillet and rice.

**a. Fingermillet neck infecting isolate (MG04) specific genes**

>MG04_T11747-R1 protein Name:"Similar to MGG_17305 Uncharacterized protein (Magnaporthe oryzae (strain 70-15 / ATCC MYA-4617 / FGSC 8958))"

MPLKAVTGGTQTKLPPVDRVFSTAPTLASQNKQPNDWRRAYRPETAALAPVDRVVQTNPP

YMGPWRRSNCQQQ

>MG04_T09650-R1 protein Name:"Similar to OOW_P131scaffold01822g9 Uncharacterized protein (Fragment) (Magnaporthe oryzae (strain P131))"

TLQRAAICVPVQTGVSPRGLNITNPITNYTPRVRGRIARSAQPDLVYRSLGEDALHTVGT

DSYQDSLQNINTMQARYACQRVRGMRHVVNSVRRMILPAMPINVALQATADKDSAWGVAA

RPRHRSRYRQIAEI

>MG04_T02015-R1 protein Name:"Similar to OOU_Y34scaffold01062g1 Uncharacterized protein (Magnaporthe oryzae (strain Y34))"

MRFFATLPFMATAVLGLATGLVDRADLPDGGYVFNHYDNGTVTALPLDTPGAEEILIKPI

VPKAEPEAEHGIQKRWVTCSPGNTLDPAGVDEGVRQLQSLHVSTARTGLDQAVAIFRLAI

EMQSYGSYHVAVMMVYYCIDTKWWTGNLDIADIDYALRNMDSVCRRYTASHFQWDSPEIV

GKADVNLAVCCGGGNC

>MG04_T12497-R1 protein Name:"Similar to AVR-Pik AVR-Pik protein (Magnaporthe oryzae)"

MRFTSFNAFFLTLGTIATVNAKIENAIVPGFRIDLSREPNPVVYDAPGSPVQECFWFMFY

NGQRVDARACHSGWSFGLQVGKRFVQIKTDKKFN

>MG04_T02978-R1 protein Name:"Similar to OOU_Y34scaffold00714g5 Uncharacterized protein (Magnaporthe oryzae (strain Y34))"

MFSTLKCASGATGYVVEPAEPPFNKRQKVLARVACAYCREKKLRCSGEPEGCQRCLARSI

ECVYLSQRASPAGHRRQSSTATLASSDISSISTLAPSTAEETSQDKLMMWGDDDVWNYTE

GLDDMQWPGAAVMNRASIVTLEDTSCTNTRATDNLGHRGSVSSAQSPIDKVSRRTTPRHS

TPLTTVSDAAFGCCDCINQLLQQNEKLSITLFTGRAERKPCPADIFTNAIWRCHKDSMAS

CEALLSCDSCSSRSELVILAIGMCRMIMTSIEDIHAQLRSNVLLDDDLRSEQSLDRNPRA

GDKRTRDDVSGAEERQRSRSPLSGQWDLDEDELHIVRGLLDSRVARIGCLLTKIRQITVA

RHWPDCETMNEGIRKRHAFIAANAHECRL

>MG04_T00922-R1 protein Name:"Similar to OOU_Y34scaffold00714g3 Uncharacterized protein (Magnaporthe oryzae (strain Y34))"

MSLPLSINIDPGFIPYLSCGLVALFLWFSIERPKTADFPLLNKKREEFDVGGIDLVRKWL

AKNGDQPVNVTADTGPFTILPTKYAKELRDKPELDFAALNYKKFHGNIPGFDAFLQGTKP

VSVKVVSTHMTRSLASIAKPVAEEVSVALQDLYPQGKDWQEVAAGELNMLLTTRATARIF

LGEGVCRDMEWIKTTCIYTGALFVAADKLRAWPAVLRPIVHWFLPTCFYTRSVLKQAKKI

IEPVLAARQQKRRQLIAQGKPTDGFNAAPEWFEQASNGGQFDYDPVAAQLFLAVGANHST

ADLLTQTMLQLALHPEYIEPLCDEIKATVLRDGWTHNALGKMELMDSVLKESQRMKPTDV

FTMRRLVTKDMTLSDGTFFPKSSNLTVSALNLWDGSLYGDPERFDGRRWLRMRRSDPARQ

AAAQFVGLAPDHMGFGFGQHACPGRFFASNVLKTLLAHLLLGYEWKLADPSRPIRHMEWS

GTLRVDPGLRFLVRRRAEPVQV

>MG04_T03357-R1 protein Name:"Similar to MGG_10614 Isoflavone reductase (Magnaporthe oryzae (strain 70-15 / ATCC MYA-4617 / FGSC 8958))"

MASSKLKVVITGASGHTGVSIMNQLLAAPAQFEVTALARTESVNKQIYKDLAARDALVKP

ANLQDVDAVATMLSGADVIISFWASVCPPTGFMAVRDEKEALFNRCKRLYLPYTVIDVGL

WYQVNIPLVPSGRLDDLIVLDDSFIFGDGNTKTGLIDIDDIGRYVARIIPDPRTLNKMVA

AFGQVTTQNEIHSIVEEVTGETIPRKYRSRKDLEETISATVEKLAQNPIDEGLIMQKFIL

GYACSRGIRDDNNLDTAKYLRYLDAKELSPDVECMSFQDYIR

>MG04_T09841-R1 protein Name:"Similar to OOU_Y34scaffold00971g1 Uncharacterized protein (Magnaporthe oryzae (strain Y34))"

MKYGSILAALISLAAPASVLAVWEGNTPRGTEMLKEGGASKEIAGYTCRAGAGCKAECDN

MSHPFRVLGTPHGEWC

>MG04_T07284-R1 protein Name:"Similar to OOU_Y34scaffold00886g2 Uncharacterized protein (Magnaporthe oryzae (strain Y34))"

MVPFIQRLLIQRREKKNLPSFYAQAVTNIVSDLAIFFLPLPALWKLHINGPQKLVLLSIF

SLGLFACIISIIRLTFLADSPDITWDNASASAWSTAEVCIALICASLPSLRPIVGGILPR

LASSFGKSTTARSATRKGTSRLQTVTTVTSRKAPTLYPDLEGSVERLNSNEGSEGAPVRS

QTFLDDGSDHNGFAMTDMAKSSAPPDNRI

>MG04_T09033-R1 protein Name:"Similar to MGG_10780 Uncharacterized protein (Magnaporthe oryzae (strain 70-15 / ATCC MYA-4617 / FGSC 8958))"

MRPCNLFATLTLALSVHANTGNIQNPDALAARSTVGGGGNEFNTNGFAKHGTGQPAANQK

SGPAGRGPSNRAAHRACHRRGGKLVPHGAGRRQGGKLMARAAVDDDDDAKSCVTDDGNSG

YYQDFGDLPGGGFDDPGTDTEPTVVD

>MG04_T11214-R1 protein Name:"Similar to OOU_Y34scaffold01006g1 Uncharacterized protein (Fragment) (Magnaporthe oryzae (strain Y34))"

HLPPPRAHPHTHPSPRPCLCPTRGGLSTNAATCRNSRETPENERAQNAVDSFWDAIESEN

MVPGSFKTMDFLFPYNGGLGPVRTTILWQVLKSPNKNRSFKWDSTLCRKYLSVPAIVAI

>MG04_T00938-R1 protein Name:"Similar to OOU_Y34scaffold00266g2 Uncharacterized protein (Magnaporthe oryzae (strain Y34))"

SFFFFFFFYPGVIRLGGVEAVDAKRTAAKTGRARPPVCPESRRQPEPDCLDTPRARSHKA

QPVLSIPSCSSADADDNPL

>MG04_T00172-R1 protein Name:"Similar to OOW_P131scaffold01596g2 Ent-kaurene synthase (Magnaporthe oryzae (strain P131))"

VTKTHDGEMQWLFPESFRVLLEEQDADGSWESSASQIDGILNTAAGLLSLIRHAKQPLQI

AISGEDLQGRIERATTALQRQLDAWDVSLTLHVGYEIILPSMLKLISSEGVEIGPWRGEA

ELMAVNAAKMSRFKPEYLYGKMQLTALHSLESFVGLIDFDKVAHHKFHGAIMSSPSATAA

FLMNLSTWDDEAEQYLRDVVARGPGSGRGGVPSAFPSTNFEYTWILSTLIQAGFTADDLK

SPELQKMASILSRSLQDNGIMGFSPGIEADVDDTAKALICLGSLGQTISPEGMIKAFERE

THFQTYANERDPSFSANCNALLALLCQADPSSCSAQILKVVQFLSNAWWISDGPIKDKWN

LCHLYPTMLMVEAFVRLLAIIDQGRLPADFLPEELRSKVAIAVFQAGLRTMMEQKPDGSW

NHSAEQTSYAVLILSESRRLRHFEFIQPDMEASIERAASFLQDAAIVPEPVWIEKVTYSS

PLLTKSYRLAALKAASTTHQAVGHGIPLDITERKIFKYHQMYDMTPMFSSTPGWLIRASL

VEGSLFLPLLRRGHLDIFPRRGMAEDKYFDIIPFTWTGCNNRNATFASAAFLRDMMRIAI

LDYQADEFMEAVAGSWFADDLGKLVEVIDHIFSAPSQADGVKAVDETSNETTSGAAQPSG

TNGLPTAPEPAEHVEARECMRRFVKHVMEHPSVQAASPADQENLQREFRQYFLAQVDQIR

HNQRFGAQDQQRRYLSPRTSFFQWLKETAARHVACPFTYAWALCVVPYVVGNLSRAREGG

GGPSDCFASVEEKYYSADLCLHLASMCRMYNDHGSEARDGLEHNVNALHFPEFARTLRGA

PSRDALFQLAEYERSCWQTGLARLQEVSLQEPDVALRRTKERRIAVLRTFMDTVDLYGQI

YVVKDIASRMVPAQPLK

>MG04_T09671-R1 protein Name:"Similar to OOU_Y34scaffold00266g1 Uncharacterized protein (Magnaporthe oryzae (strain Y34))"

MGWTYRTFGDTNRYYTTQMLRLLFDDFHIQTVSIYSVDNVYTPREMTPEQIEWLKNWRAW

ELCPSDPPPWPDRKSDDFKDEGGPRQSLHCERKGIFGTLLDFCLPRRLTGSK

>MG04_T11790-R1 protein Name:"Similar to MGG_17475 Uncharacterized protein (Magnaporthe oryzae (strain 70-15 / ATCC MYA-4617 / FGSC 8958))"

MIDRTHGKEKTGSPTSCHILPQFYRRRASSIAKAASLDRGRAWCGTYLAFFGGGRRLWLP

IRDRSTRATSELCCRSITYRPK

>MG04_T02730-R1 protein Name:"Similar to MGG_05423 Uncharacterized protein (Magnaporthe oryzae (strain 70-15 / ATCC MYA-4617 / FGSC 8958))"

MQQVTKVNSMFTQHEAMSSGMNAENGRRIAFMALLYLPISAVAAIFAMPIFKFENDWRDI

YLRPVPRSKDDEVSDGMPVVSGYIWYYVVISLVLTCVTTAILLFGHRLKRRQDRRTEGVT

LGAVKAGK

>MG04_T04704-R1 protein Name:"Similar to MGG_05011 Initiation-specific alpha-1,6-mannosyltransferase (Magnaporthe oryzae (strain 70-15 / ATCC MYA-4617 / FGSC 8958))"

MSIKLADFSVASSLCDQAVQHVLRPRHRTRLLAVLALAVLTTCWLLYSTSSLQQTGQPRS

HDLTGTKDHSTWDDTSAWPRLDWVKAHAGILIPANIWQIMLPKVWGGKFVVNSGTLKETS

TWLAMNPDYTYTLVGQDGGKDFVNRHFGHNETIVSTFNSLPNVGMKSDLLRYLILLVEGG

VYTDTDTVALKPIDDWIPKPFRSKARLVVGIEFDQRDGGRWADIPHALQFCQWTIAAAPG

HRVFQMMISRVIRSWQDLKQKHNITNDAGSTSMKLTNFEVLNSTGPAAWTDVVWQHLQEI

DDTLTDIRNLSSLGPPKLFQDVLVLPIDGFGVGQPHSGSTNDGSVPDGALARHLFGGSWR

GD

>MG04_T08109-R1 protein Name:"Similar to OOU_Y34scaffold00710g2 Uncharacterized protein (Magnaporthe oryzae (strain Y34))"

MSVNANHATTFGTETSGTVVELVDTTERLADVLSSLTTLPTSPPSLYVDLEGINLSRMGS

ISILQIYAAPLDHVYLVDVHKLGSDAFTTKPRGSAGQTLKQILECPWIPKVFFDVRNHSD

ALFAHFSVGLQGVADLQLMEIASRDRWRRRFLSGLAKCIELDAGLGLGERSAWVRNKDRG

KALFAPELGGSYSVFNIRPLPDEIRDYCSQDVQLLPKLWARYDARLSAAWKDRVREAAVA

RVGESQSAWFNGKGRHMALPPAGWDRLD

>MG04_T03124-R1 protein Name:"Similar to OOU_Y34scaffold00714g7 Uncharacterized protein (Magnaporthe oryzae (strain Y34))"

MSTVTEPYMACPIETDTHEAYSSPLARDESPSSEHGSWSSAKTFGSDCASEITQASTTDG

HEQEYNDITKHLPSVSVPKEVLHVDFTGCSLTPVMTPFNYVSSLPSKGVREQAIMALNDW

LDTTPESIQLVTTLVADVHNMSLMLDDVEDNSPLRRAAPSTHNVFGMPQTVNSATYMIID

IIGRASKLENPRILPVVIDEMKNLVAGQGLDLYWTYALSPPTVQEYLDMVDGKTGGLFRM

ISRLLVACQRTPQESPDLNKLMTLLGRYFQIRDDYMNLVSHQYTDSKGTCEDLDEGKYSY

VMIHALENAQPNTRRTLQALLQQRKSTGSAGPGQKDLFLQLFWEAGSLEHTAALLRSLGV

AIAAEVGQVEQATGRTNMGFRKLLEKLKV

>MG04_T05900-R1 protein Name:"Similar to OOU_Y34scaffold00714g4 Isotrichodermin C-15 hydroxylase (Magnaporthe oryzae (strain Y34))"

LPCSKVWRAWAWMSGRWPYLVQDLHDKYGPVVRVAPNELAFFNVEAYKDIYGHASRGKKT

FIKTEFFNSGEEEGIATVKDPAEHAKQRKMLSRGFSQQSLREQEEVVHQYVDMLMEQLGK

LGAPSGTGINLVDAFIWLTFDVISDLAFGKSFNAVASGKTHFWISLIFEAAYVSQLADLR

RQLPLLILLLPFVLPKGIIKKGKQHNELSRAMAVSRLKQGDTGRADFFSHLLRHSGDGTM

SEAQLQSQASILVVAGSETTSSFLSATCYLLLKNPDALARLRREVRSAFSSLDDISADAA

AALPYLNGVIEESLRLAPPQSFGLPRYSPGAVVDGHYVPAGVTVSAEPFPMTRDPRYWKE

PDSFRPERWIGDGFGDVREASRPFSLGPRACLGINLAYMEMRLVVAKMVWRYDWELMDTE

SDWFRGSRLHLFWKRPALNVRFHPRA

>MG04_T06097-R1 protein Name:"Similar to OOU_Y34scaffold01048g2 Uncharacterized protein (Fragment) (Magnaporthe oryzae (strain Y34))"

KAPEDDAIAIIGLSCRLPGEASSPESFWNLMVNGRSAYSETSTRWNTAGFYNAAADDRLH

TTKAPGGCFIDQEHSEFDANFFRISQQEAAALDPQQRLMLEVSYEAFENAGIRMDQLSGS

RTGCYVGVMGTDWKESFSRDPEAAPKYAYTASAPEFVPSRVSWFYNLAGPCMAVNTACSS

SMVALHLACQSLRAGECEAALVGGVNVMINPDFSCHLSGQNFLARDGRCKTFDAAADGYG

RGEGCTALVVKRARDAVRDGDSIRAVVRGTGLNHDGRTKSITLPSREAQAALIRSTYKLA

GLDMGEASYFETHVQSTKQWVSCAKSKTPSPSVESSQISGTARR

>MG04_T02244-R1 protein Name:"Similar to MGG_14244 Uncharacterized protein (Magnaporthe oryzae (strain 70-15 / ATCC MYA-4617 / FGSC 8958))"

MWTTRLLSLLLLSLASKGCLGQSSAAIRGSVTQAELDTFKLYADYTAAAYCNKSPDRVSQ

KIVCGSDACPLIEAHETTIVATLADDGDRAGGYVALDSTAERIVVAFHGTITFAGYMADF

NALLQDDDLCQGCQIHAGFRSIWAAVGDVVMETVEKLHSEYPDYSIVTTGHSMGAALATL

AGANLRQKIPEKVIDVYSLGSPRVGNQAFAEYVSAQPGSVFRITHVNDPVPRLPPNLMGY

YHTDVEYWLSTGGALTTDYTPNDVLVCKGIFNRNCNTKSDFFGFGFAAHVNYLTRIGACK

P

>MG04_T02038-R1 protein Name:"Similar to MGG_15515 Uncharacterized protein (Magnaporthe oryzae (strain 70-15 / ATCC MYA-4617 / FGSC 8958))"

MPDKIEDMYRQMWSKLAKDQRDPRPARAALYFKMCMIEKDHSQQYFLNLLLVALGSTDHK

LHLPAFEDNLIDRLGSLLGTCEFIEENIDNDSLGFIDRVYRGEEEAMLFRMHREGYMETI

AFGPVNLVWGNETHKGCEELIRYANDDMVFRFVHRSVYDFLTDTEEGTRLLERCQLSIED

IHEKLIEVSNLRFSTRPDDAREKLVDALATRFEYWTDRIVLLHGCIHPADRVQFHEDTFS

MVTPLSITLADGVNAFYEDTYKQRHTGVVQTSRPAWTYFLARCQFFLDGECSIHDEILLW

IGHPDSEAFQTTYKAKARFLRSYIDHRYGLYGMENQDTIAICVLSFPIALVLRYILQALK

>MG04_T11948-R1 protein Name:"Similar to OOW_P131scaffold01008g4 Uncharacterized protein (Magnaporthe oryzae (strain P131))"

PDFDAHNEVYSADKIVRVEFDSPYVKGLPTIKTVREKARGRGGGFQERRVRETYTDAYTL

ELEELHRCVVEARSKTSAVDARRDVELFQMILRAGAAKLEG

>MG04_T04681-R1 protein Name:"Similar to OOU_Y34scaffold00714g1 Uncharacterized protein (Magnaporthe oryzae (strain Y34))"

ESIGAGRVLDPEWIDIDLLRGWKHECLTTHGDKCRNPFGIPQVSPAWLIDTAEQRLVRST

GKHVDFVALSYTWGTGVNSAFKTETRNIRDLELAGALSDLPLSPTVRHAMGIVRALGERY

LWADALCVVQDDQVHGAEQLRLMGGIYASAKLTIVAADADAAVGILGIRDTSPARGFDQA

CAPSFGIGGVQRIIVRRNPLERMLSGFNSYFERGWTYQEFFLSQRRLIFNGLQAFWGCSP

GRKSEDVVGPFPNNSDWYITDDHLAQILNGHPDLASLSAMFSEYSRRDLTYPEDALPAIS

GLLTLFERAFKGGFLYGLPEVCFDAALMWRPINIASTKPRKSSGKTKINAFGLTSALPTW

SWLSLRTFGLDTLPTEPFVGWRRKPLVIPITQWYTHETARHPHKRAIRSSWHMTAQRADR

QELWQREGWVREEFDASRHVLDGDRVGPLGRKAPPEDLGRYIFTHPSLSGKEYWAPVPFS

AKEPRLPSPPQTPYISCQAKKGRFAIARRMSKYGGYNHLVMVGADAVGILHVHQESDSEL

FPMAHHADADKVMVELVAICQRRRLAAQVLDYELDVNCPDEYGVLWVEWRGDVVAYRRGS

GYIGKEWWDGHDLEDVYLILN

>MG04_T09873-R1 protein Name:"Similar to OOU_Y34scaffold01062g3 Aquaporin-2 (Magnaporthe oryzae (strain Y34))"

MDETTPQHHKGAMLGHADGQAGPHRQTLRHHLVAALGEFVGTFLFLFFGYASHSMIATAE

RPTPEGLRDGYSAQATVFIALAYAMAILVTVWAMYRISGGLFNPAITLGLGLAGQLPWIR

IAVLFPTQLVASICAGAVIEAMLPGPISRVNTKLAPDVSVTQGVFLEMFFTAYLLFVVLM

VAAEKSKDTYIAPVAIGLAAFVALIP

>MG04_T08248-R1 protein Name:"Similar to MGCH7_ch7g68 Uncharacterized protein (Magnaporthe oryzae (strain 70-15 / ATCC MYA-4617 / FGSC 8958))"

MVSFTTFLACLTFTTVALGDKTTPPSKEFVVYYSKPGDGMAELYAGRRKIEAALESQ

>MG04_T00412-R1 protein Name:"Similar to MGCH7_ch7g1076 Uncharacterized protein (Magnaporthe oryzae (strain 70-15 / ATCC MYA-4617 / FGSC 8958))"

MSSFLLMILRSIDPRRRQKSDRAESAPKWRPTDTVLPVHAIDNTATLKAMVTSQALRFND

VLDGDLLHSSLTELLSTGNWKRLGGRFRVNSGRWTSNSLVLQSANMLELHVPDRFSEAVP

AVSYTHAEYPGNLDETEDAEVLQTHAGKARVVDIPDDARRFLVGHQTPMSLDDYVSRDIP

LINLHVINYADATLVTMTWPHALTDGMGWIGIVRNWCKVLAGLKDEIDDMGGLHQDPLAP

VAADDGPDRERFALEHKVLQGLGLVMFSVRALVAWIMSPQILHKGIFIPAALFEHVKQQV

TEQQEALYKDDAENKPFISNGDIFCSWMVRMACSATFAMSTRSVVLMSAVDIRSRLPSLF

KPRTVYAQNLLAFSFTFVPLGDVLRGPLSGFARQIRESLVRQTSEAQIYAGVRRNLRSIS

ASGRMPLYADANSILVVMSNLTKLSWHDVFNFKPAVVGRGPGGRKETAGLPVWQVACSES

KDSYATSLMFNGADLDGNYWAEATLPAPAWEHLEKQLVEWERSAMKTK

>MG04_T00413-R1 protein Name:"Similar to OOU_Y34scaffold00092g4 Uncharacterized protein (Magnaporthe oryzae (strain Y34))"

MDSTASTAHYPRWMAGLLVAHYAVVSLSILGLSATSKARYASVCLLGAITLALQKTFMEL

SDSPFTSASILPCLTIQFMSSSELIIISRAVGTDFYPGASPHSLAARVYRVTLTVWNLRR

IGTKWQIANIPPYSAPRSRIVYATRTVAQSALTYFLLDAMSALSPTDPVFYSVPKQTLGL

DVLKLSWQDLAFRAASTASFWATIYCAVVLQTNSVSLLVVSAGLDDAASWPPFFGSPWDA

YSLRRFWGHFWHQTWRKFLTGHADAISDKVLGIPRGTTVSVYTRLWLAFFISGVCHISSD

VGMGIPFSKTGAFATFLLQPVGIVLEDLLRATLGKVVPVPGGVKRVLGRVWVLAFLAYST

PVWFYAQHRVAGDSGDALPVRVAPRLLSCIMQHFSG

>MG04_T06756-R1 protein Name:"Similar to MGG_01900 Uncharacterized protein (Magnaporthe oryzae (strain 70-15 / ATCC MYA-4617 / FGSC 8958))"

MKELATLLDPGSLSAPLLGASAAAIITDVSSPEVSHVVDDYDDGNETPHGRHRTATEGAN

RSRWEVSNIRNIVKDANPGTGGSCRLVVFRGSMIRYMFGVGDGGFEWRVDRRDC

>MG04_T00713-R1 protein Name:"Similar to MGG_07917 Uncharacterized protein (Magnaporthe oryzae (strain 70-15 / ATCC MYA-4617 / FGSC 8958))"

MAETVGLICSCLELTGHVFEVAKQVKLIKNAPKDIRQKHKEIAERASKIMCIMQILEKSG

IHAKSILLSSQPLLDSIHEAKELLERVFPRGQERKWELVRGGIKAVWDKEEIKEALDKVH

QEQMVWLGGLPAHLHVQVMYVHAILGAFPRLISASLVINLLEYSEKLDKVSEGHIRAEAL

GVETLASTARIESAVAGAQEFVEQAKKEFMDEMRLLRQQFYNQAAVDINQKVPANTITNS

NVNESTAVVLAANKRALDNLDSKDCNCVRLLTRRAIMKASIVSQSLYQHRQDCPYYESGD

MRQTYCAYLPSWSVCWLPGLRGKYVTVGWDHRFKGGAFSTYPILRITRQIRRGDSPVFKV

LDQFSASFPWAMWAWKTEFPEIHQRVAKPEDLEASIDRIKSFFRTGKGFPTDVDENGQSI

WQASLMIFKAALIGEHSELIPSLRELLLTLLESGVQPDQEYRGSDYLTLKSIWRWPIDQA

TYEFFDGKTTALDGFRLPLYDELKAAKYIEDDAKFDSIFEKIPPMAWRPLLRAHPELLDD

YGLTPIAQRLLLTSTVEKFKDSVTCKSLRDWDRENNWWHKRVLLGWPEIIPYIAQAGCSL

PIALEDACLTGCVEIVRALLRVEYLPIRTVHLDNAFNCGSSRVLETVVQEMMARREMVCE

LASAVLQPDVLEGFGLCVPGRAPSPRDARRLLEITLDEYQGQLAKALRNLGSSTAYFMID

SEFRDEQTDEFAMVERLSCINLFEEYTFLDPASCNLGGDYAGAIQCLLDAGFHDLDREDD

NKQTVLYKTCHNYKPEGAEFGVDDRDLLWLLKHSKKNQFPLELASEHQSLVSPIFYAASA

LRHVSIEALKDAGVLEQLSEVPTDCCECFCSSNGCMPHYMFLRCDGNGCQEGAKHDACTG

DAYGVTGRDGCLHQWCEAWALPDHQMELYYAEACRLEVFERLGMKHTCCASGRRKDYLDR

IRGLEGIRSRFDDYQTERPRVNIPNRWPRADDSERQEFQEEDAELNADLERVMHYYDKMR

APFLTSPVCGEADEDFCDSFWFLWWSCVDRILPPLGKEKCVYRGIEFSRREIYKERGVYE

KMDEEFADKREDEREAALSAAGYEGLDFRDVVDEYFGGSLDAIVFLRSWRAN

>MG04_T08281-R1 protein Name:"Similar to OOU_Y34scaffold00608g93 Uncharacterized protein (Magnaporthe oryzae (strain Y34))"

MRASFIAVAVAAVAVQAAPAVDARQAAPAIPAACTPTPGLDTAATYDKVFAFQKAYLFDK

NVAETFKYYSADFQAIQRFSNLNRDQYWATELVQQWATVPSYPTDSSFRDGRAHVAYDLG

AERTGHAGDDFVWKDGCIVSQQQTRS

**b. Rice neck infecting isolate (MG02) specific genes**

>MG02_T12598-R1 protein Name:"Similar to OOU_Y34scaffold00875g6 Uncharacterized protein (Magnaporthe oryzae (strain Y34))"

MCGSIAVWALGGQRGQFLCNSTQKVWSYPQLSQGQGARTTTTSAPLNEDGGQPEITPENR

TIYCPHKRWDEVYNLLNNHFNTNINHSQYFADWTSTDFARMKNLHPDKTMPEVLEAMLDK

LQLVQRALGPGFQGEVALYTAVARACRGVKELENALLTQKPTCEALFADLRASLQVAMDH

KHDNAYLTDQPSHDVNFTDRRYFSNNNTNRATPRTPYRPGQGSYIYNTPRPREETRNNAP

SRTNGSKRCYVCKKEGCWSSNHPRTDQDKGRRIYIQSHEALGQEPGDYNIFLSEYEGTPP

SQSYEQDDWQEENDIQEVQV

>MG02_T11939-R1 protein Name:"Similar to OOU_Y34scaffold01043g2 Uncharacterized protein (Magnaporthe oryzae (strain Y34))"

MLESAAASPLNNKYYGEENIFRLAAILASRMIKNHAFQDGNKRAALYSADMFLKINGYQL

QKKPMAPDDEELNQALADAHVAVATNVWTK

>MG02_T11759-R1 protein Name:"Similar to OOU_Y34scaffold00605g3 Uncharacterized protein (Magnaporthe oryzae (strain Y34))"

MEGPGISSRNKNAAGNNAPQDAALLDAAPQETAAQSGGESATKLQEGNSGTDLRPLNIQR

SESGNLPASLGVPSTMQNNFQEDTPMTGTSPDPAADSLNYQTPTEPTKEQKLYYVDERHF

VSAFTSVPSVKKTHEAKGWDTTIRLRGRTTYRLKKYYGIYWLEKSKHKLSECQGRFEDDK

EKAKLEFEGVNALAIEYKPDLWQHRFDMDYFFNKKKLEEEQKWGPADAMGYAS

>MG02_T11758-R1 protein Name:"Similar to OOU_Y34scaffold00605g2 Uncharacterized protein (Magnaporthe oryzae (strain Y34))"

MLGKNIEDAVYDTVAFAPIGPYGVRVECTYMKFEARSDHNCGPAPVGPGAFAYRHIEESE

VCTTLIRVAGPDTWETFKIDATADKWRVVKVLVANSNLSPRDSITVVSGVRMNHDLGGGP

TVGSVGDITTDADWSLAEAFH

>MG02_T12219-R1 protein Name:"Similar to OOU_Y34scaffold00109g3 Short chain dehydrogenase/reductase family (Magnaporthe oryzae (strain Y34))"

MSAPDKLFKVPGLVAVVTGAGSGIGRSIARALALNGAARVYLLGRRLDKLHETAVCPELT

QAATKAMIPLQVDITSKDQLEAAAAYIAAETGFVHLVVANAGVLGPDPLCTGVANRSPQQ

LKEYIFKNWHMDEMLKPFETNVMGVFYSCIAFLDLLDKGNSDANRLPGITSQVIVNSSVT

AYMKGLIGAGGVAYSSSKAAVNHMSKCLSAHFIPYQIRVNLLNLGIFPSEMTAALTSSES

CPYTPSSIPAKRFGEEEDLVGATLYLASRAGAYANDLSLVLDGGMLGKLPSTY

>MG02_T12144-R1 protein Name:"Similar to OOU_Y34scaffold00582g2 Uncharacterized protein (Magnaporthe oryzae (strain Y34))"

RPPKWSPLSQAREIPDDNGKYGIGSSISTRKEFRDQNAARFPRHPMEPAVLAALTMTPNL

CSEQIIDVVACGSTMGNLLRFVTSQDKQFRILVEKVGSTIFLVRRENSPTELIPDLQGYG

HSFPEHYTIWEHDVAGSKSHQRIVRYRFGSMNFMMRFEGDGFIAKTAALENSNSSSTKVN

SREKTSLDELTDSLSETTLSRGLNPPCTSLKVKRSGDLVDQDLIFDLKTRSSKKIDQIED

IIKGEIPRLWLAGISKLILAFHGGGVFRDVLIEELSDKMKEWEAANKVPLAKMAALIHRI

GDIVRGRSDGKLELCHSQAGVLEIREQLPDAGDALSPAVREMWLESTLQSDDDDASSGES

GSGDESSGVRIDWDEGEQMDYTACSTETQQRIRPEDDIIIRPSEMPQSVVQAGTNLSNEA

TAANCFPGLRVTPRVRKRGKARRAVEGCMKQFAGQLVICSSGKKPQEADLAS

>MG02_T12145-R1 protein Name:"Similar to OOU_Y34scaffold00582g1 Uncharacterized protein (Magnaporthe oryzae (strain Y34))"

MFSFLDSFVAMVEIHELTLTDTSMRCDCPVWSAFVAAKPNGEGLARRNGRQHTSTATPPS

LPKPPDSK

>MG02_T12712-R1 protein Name:"Similar to OOW_P131scaffold01023g5 Uncharacterized protein (Magnaporthe oryzae (strain P131))"

MGGYGAATVLGAVQAVGGAVTALATALILRMESWVAADCTSDLYYHETSQKYDPRGGKIT

KYVQLEILATSGGSIPLVLFVLISLLFLFLMSLCP

>MG02_T13083-R1 protein Name:"Similar to MGG_14958 Uncharacterized protein (Magnaporthe oryzae (strain 70-15 / ATCC MYA-4617 / FGSC 8958))"

MMESEHKITSTGTGPQKLDEQKRTGHGKPEVSGPWHVMAAYLTGNSPELRELNALRKRGW

ENPEGDQFFQRQRKNADHPSAKNAKFFYGLMQKIGDDLQKAVFAFADFGDSFEQKVI

>MG02_T10244-R1 protein Name:"Similar to OOU_Y34scaffold00582g3 Uncharacterized protein (Magnaporthe oryzae (strain Y34))"

MKTELRREHLARWMKAMTMESSERVAELVHTMDSAQDELDGLSSERKAETLQSVRIIGCT

TTAAAMQMKLLRATKPDVVIVEEAGEILESHVLAALMPSVKRLILIGDHKQLRPKVNNYA

LTVEKGDGFDLNMSLFERLVIQGYPHSTLQRQHRMHPDLSLYPRALTYPNLLDGAQTAMR

PAIEGLSDRIIFVNHEHPEATDNHIADRRDAGAKTSKSNKFEATMVLALVRYLAQQGYGT

DKMVVLTPYMGQLRLLHDTLIKDNDPVLNDLDSSDLLSAGLMTFAASKVKKRPLRISTID

NYQGEESDIVIASLTRSNSQGEIGFMAAQERLNVLITRARNCLIMIGNMDTFMNSRKGKA

TWVPYLELMKENGHLYDGFPVVCVRHPETKAVLKTPGDFDRYCPDGGCAELCDLPFYCGL

HKCKRRCHRLADHSQTDCTQLVEEICARQHKRRIPCGKRKSTCEKCLEEDLEAERRAKRD

LDLERSRRNRQSEYRQQLQDIQDQVAHERRVMQELLDEKKDREELEKKSRKLAAAEEVAK

EASGGSEPVSFANDSPSQTRDDWEYLKNTEGAQSPPLDELMGMIGLEEVKQAFMDIKNRV

DTNVRQNVSLQKQRYSAVMLGNPGTGKTTVARLYAKFLASIGVIPGIKFEETTGAKLANM

GVSGCQKLLDDMLDDGGGVMFIDEAYQLTSGNSHGGGAVLDFLLAEVENQSGKIAFVLAG

YNKNMESFFAHNPGLPSRFPYEIKFADYTDDELLHILGLKINTQYNGTMVCEDGLYGLYC

RIVAKRLGRGRGKEGFGNARAVENSLAIIAQRQATRLARERRQGHKPDDLYLTWEDLIGP

KPSEALSRSSGWQDMQKLCGIHSVKEAVRSLVDSIQQNYQRELDEQPPIEYSLNKVFLGN

PGTGKTTVAKLYGRILVDLGLLTKGEVVVKNPSEFLGAALGQSEEKTKGILAATVGKVLV

IDEAYGLYSGSAQDPYKTAVVDTIVAEVQSVPGDDRCVLLLGYKDQMEDMFQNVNPGLSR

RFPIASAFSFEDFSNQELRMILDLKLKQQGFGATDQAKNVALEMLDRARNRPNFGNAGEI

DVILDATKARHQSRYTKGLAKSATMLEALDFDEDFDRSTRSETNIAKLFEGTVGCEDIVA

KLEGYQSTVRTMKELGEDPKESIPFNFLFRGPPGTGKTTTAKKMGKVFYDMGFLATANVV

ECSATDLIGEYVGQTGPRVLKLLDKALGRVLFIDEAYRLGEGHFSKEAMDELVDSATKDR

YYKKLVIILAGYTDDINRLMSTNAGLSSRFPEVIDFRSLSPPECMDLLKSQFATKKSSLE

LKGITIDLTSLDSPAPDFSAAVMGMFATIANQPNWANARDVLTVAKKTFERTTRDKAGLA

AKRLVIRPEDMLLELEVFSSERASRATSTAVTGGLAGGVLARDIQYMQAPPAHQHMFKTT

TATKVATASPTTTTEEPGAEEGQDQPAALKAAPLISPDDRSASSGLYAKRDAGVSDAVWE

QLQRDKREEVRREAEYQSLKKQAEEARDADRDAIVTRLLEDEDRRKKQLALKEKLARLGM

CPVGSWPHRMFIHAGKEQSSLPLRWLEG

>MG02_T08849-R1 protein Name:"Similar to OOW_P131scaffold00581g4 Uncharacterized protein (Magnaporthe oryzae (strain P131))"

MECRPGAGGQQFSVKVSCQIEITVEWAYGAKNSNQGIDAIEGAGKVAVNDGLERPRQPYS

QQKENDCGKEQDLGEGIGIDVDINQNNQDCYMTNSSFNGEHDVRTEAAMSLGEDGSDGHL

SYEPMDVDT

>MG02_T10816-R1 protein Name:"Similar to OOW_P131scaffold01671g8 Uncharacterized protein (Magnaporthe oryzae (strain P131))"

NRLSIHVRPSDVRLNPRKNDPYRWKFIPGEEENFRAIFAKNLSEHSTGVYQLLSYAVGRR

FEAVSVQDTTPPFQLATALPCVDTSFTSVIENLSEENARLRAEVEDLKGRLSVETETRKF

IEGENREMQISQANMQIRVEELSAYTGSLQMSISSYCRGMDQVLPLLENLKTVGTEDELG

KL
